# Supplementary figures and images for: Rolling circle RNA synthesis catalyzed by RNA
Source: eLife. 2022 Feb 2;11:e75186. doi: 10.7554/eLife.75186 (PMC8937235; doi:10.7554/eLife.75186)

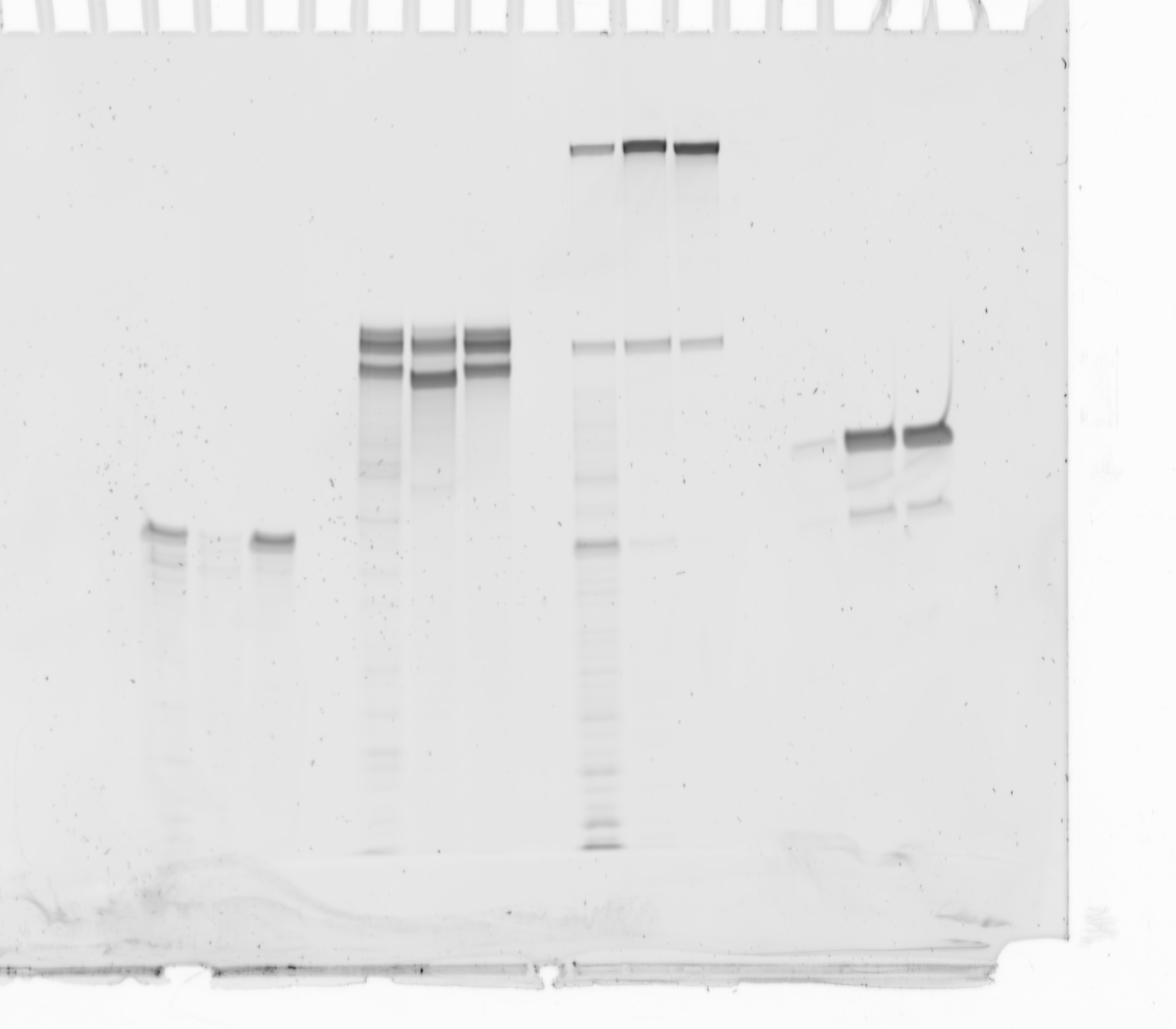

Supplement: Figure 1—source data 1. [file elife-75186-fig1-data1.zip › Figure 1-source data 1/original gel files/20181218-175115-[Cy2].gel]

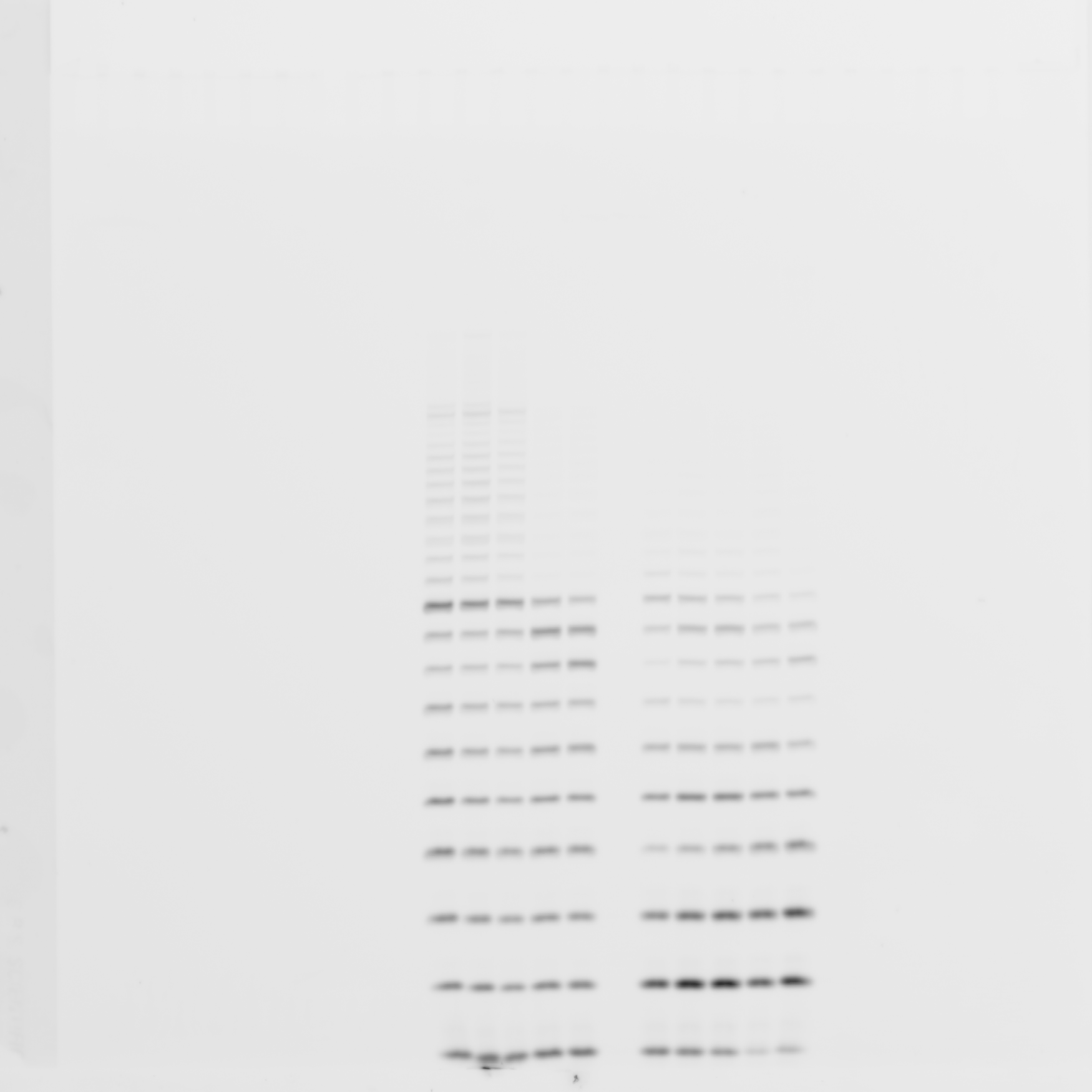

Supplement: Figure 1—source data 1. [file elife-75186-fig1-data1.zip › Figure 1-source data 1/original gel files/20201218-141135-FITC.gel]

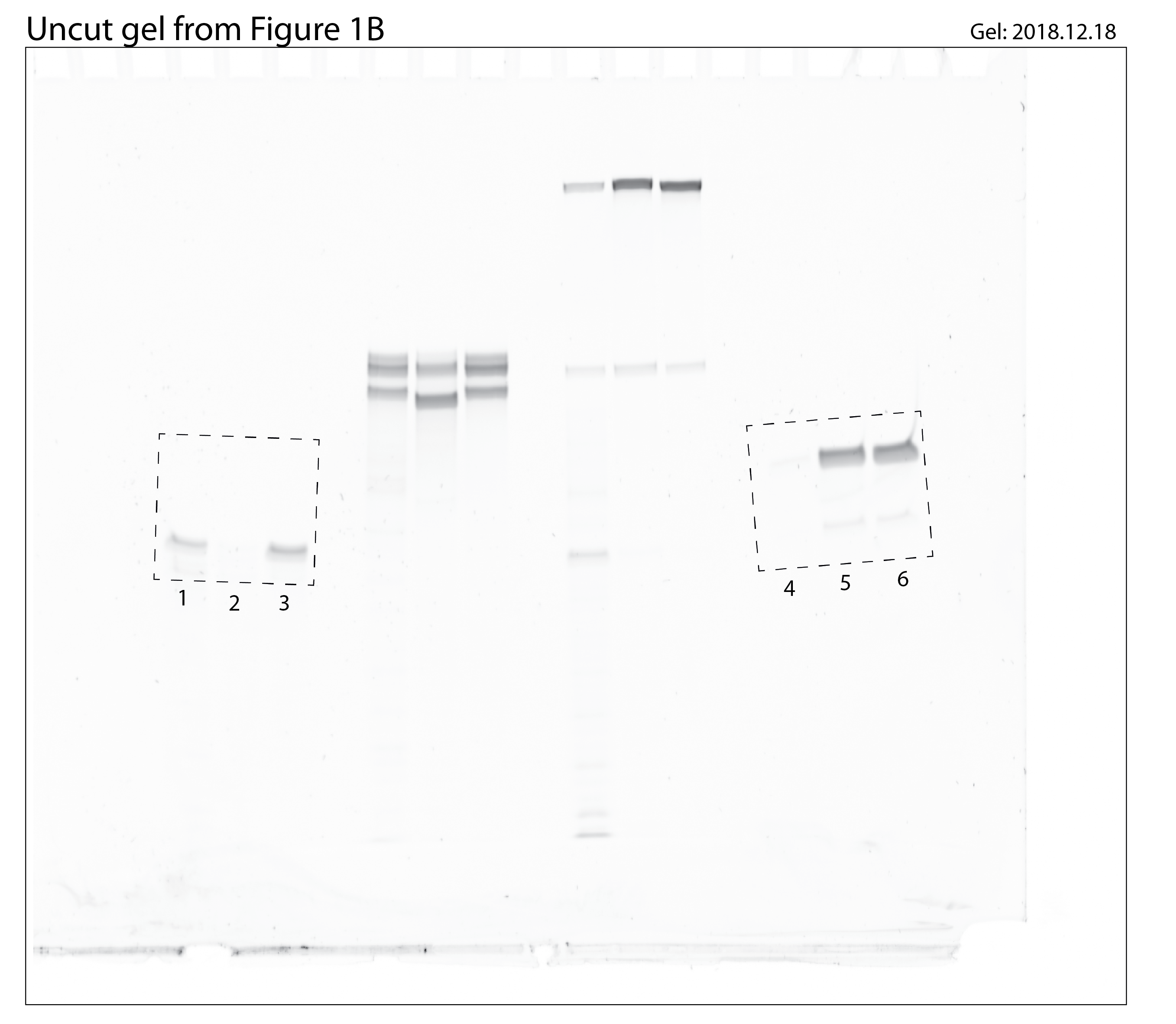

Supplement: Figure 1—source data 1. [file elife-75186-fig1-data1.zip › Figure 1-source data 1/Uncut gel from Figure 1B.png]

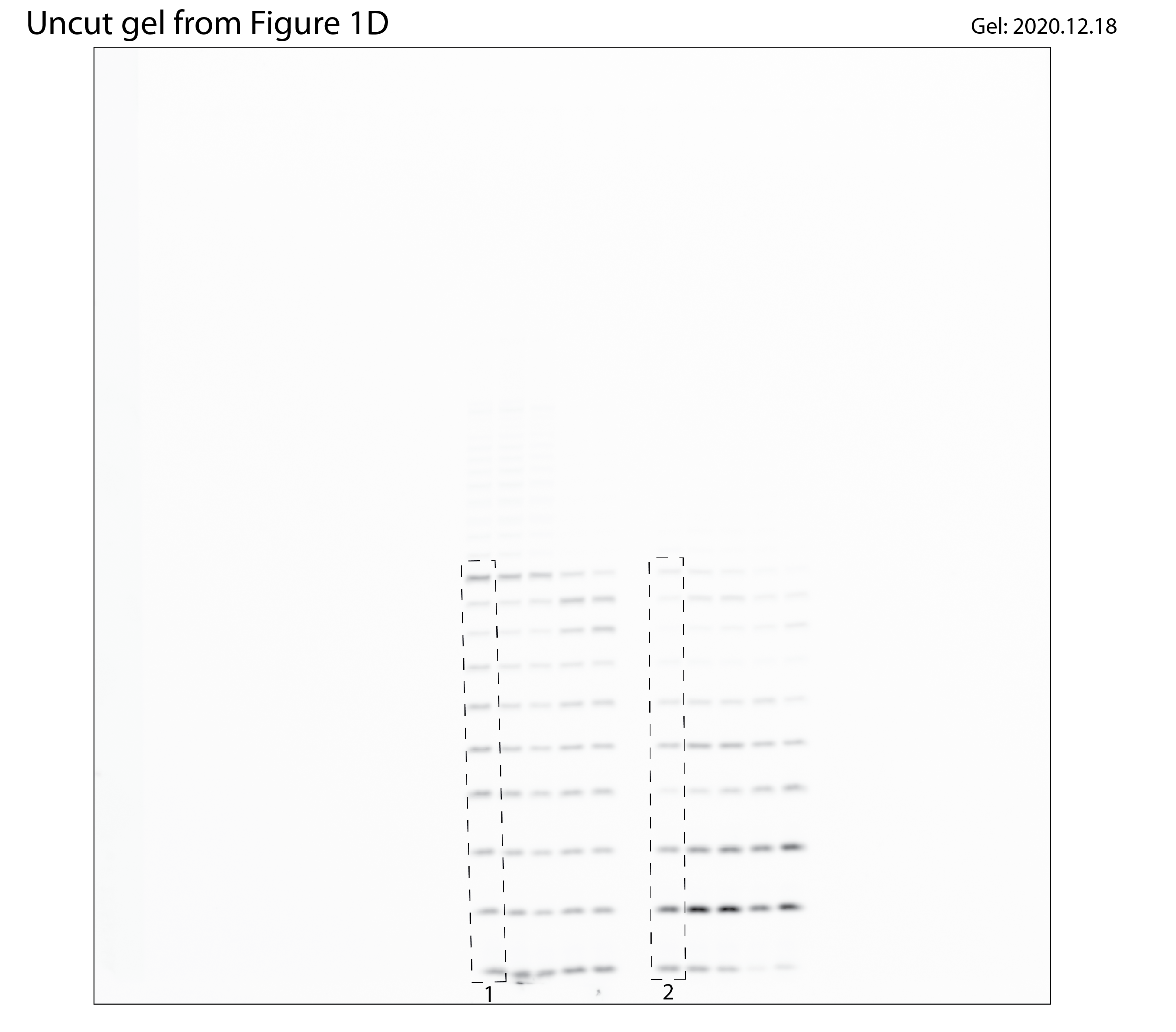

Supplement: Figure 1—source data 1. [file elife-75186-fig1-data1.zip › Figure 1-source data 1/Uncut gel from Figure 1D.png]

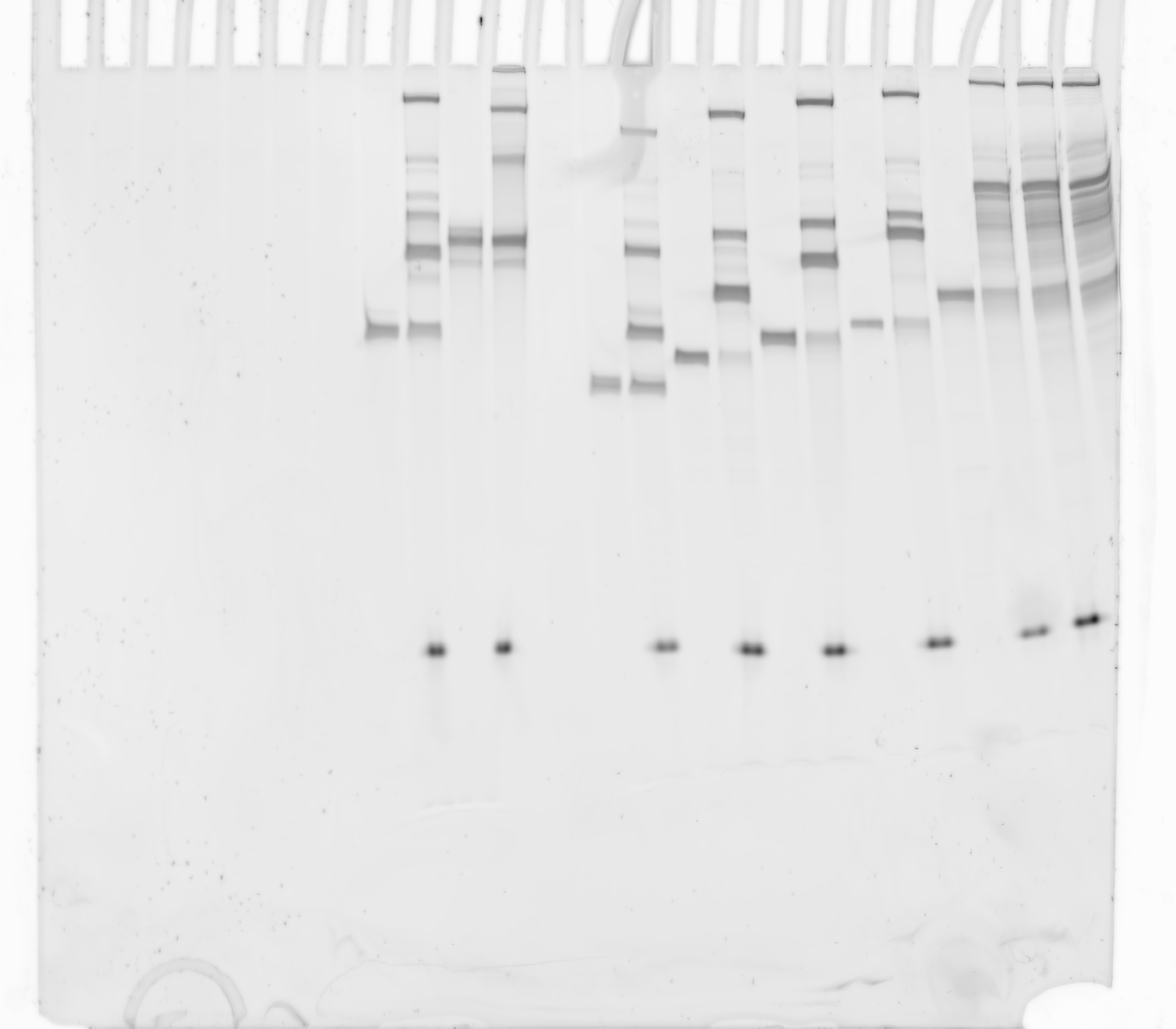

Supplement: Figure 1—figure supplement 1—source data 1. [file elife-75186-fig1-figsupp1-data1.zip › Figure 1-Figure supplement 1-source data 1/20181030-163802-[Cy2].gel]

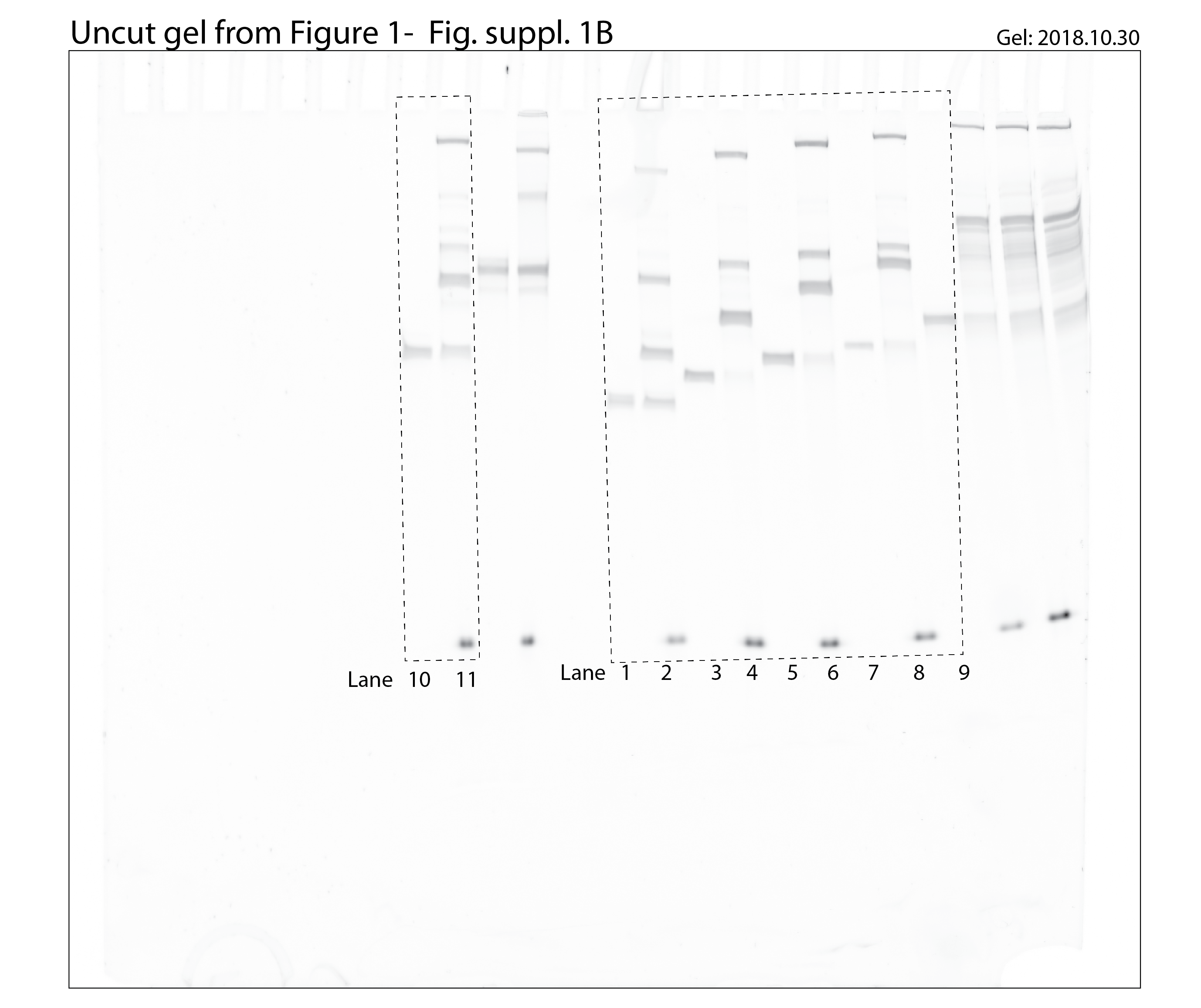

Supplement: Figure 1—figure supplement 1—source data 1. [file elife-75186-fig1-figsupp1-data1.zip › Figure 1-Figure supplement 1-source data 1/Uncut gel from Figure 1 - Fig. suppl. 1B (Gel 2018.10.30).png]

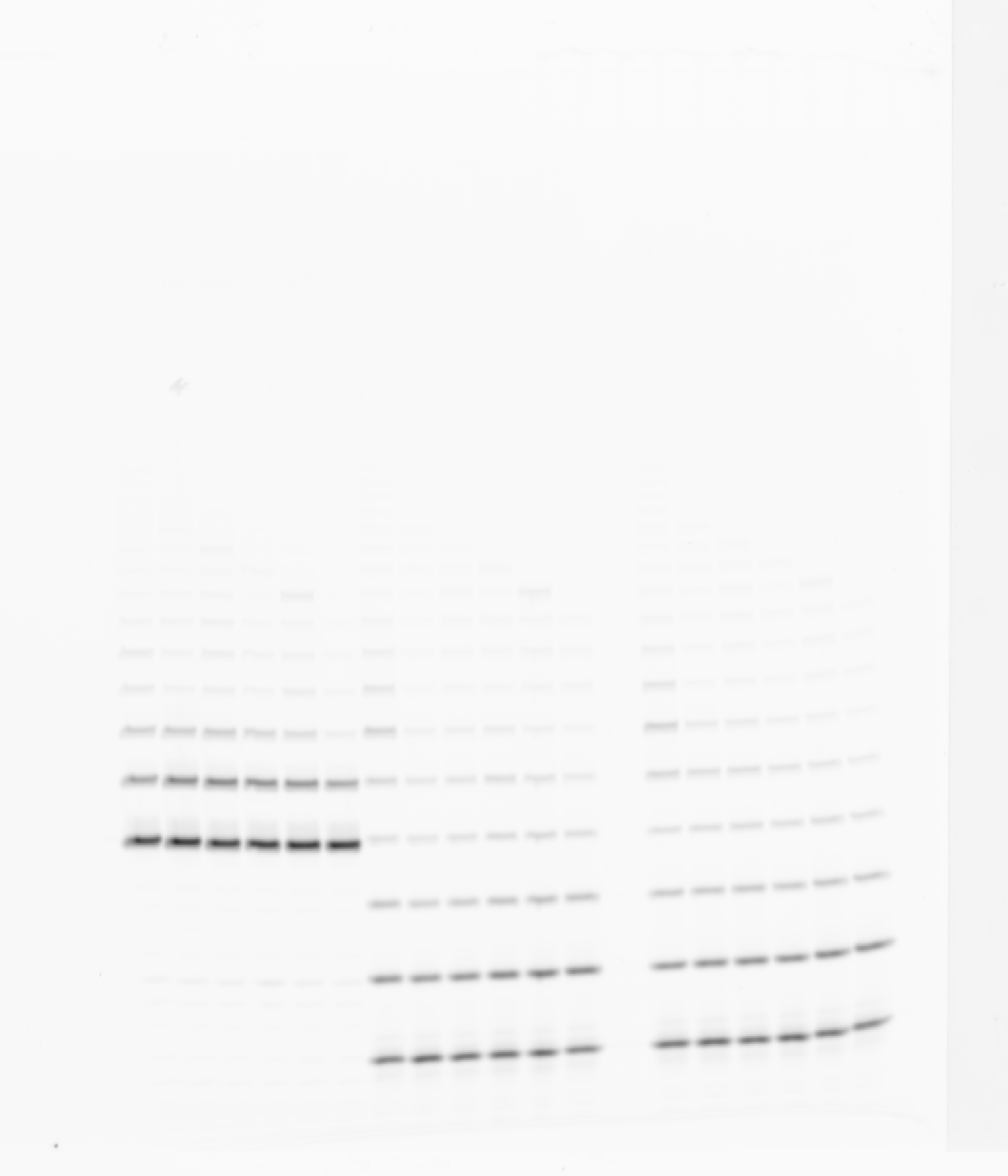

Supplement: Figure 2—source data 1. [file elife-75186-fig2-data1.zip › Figure 2-source data 1/Original gel files/20190211-144244-[Cy2].gel]

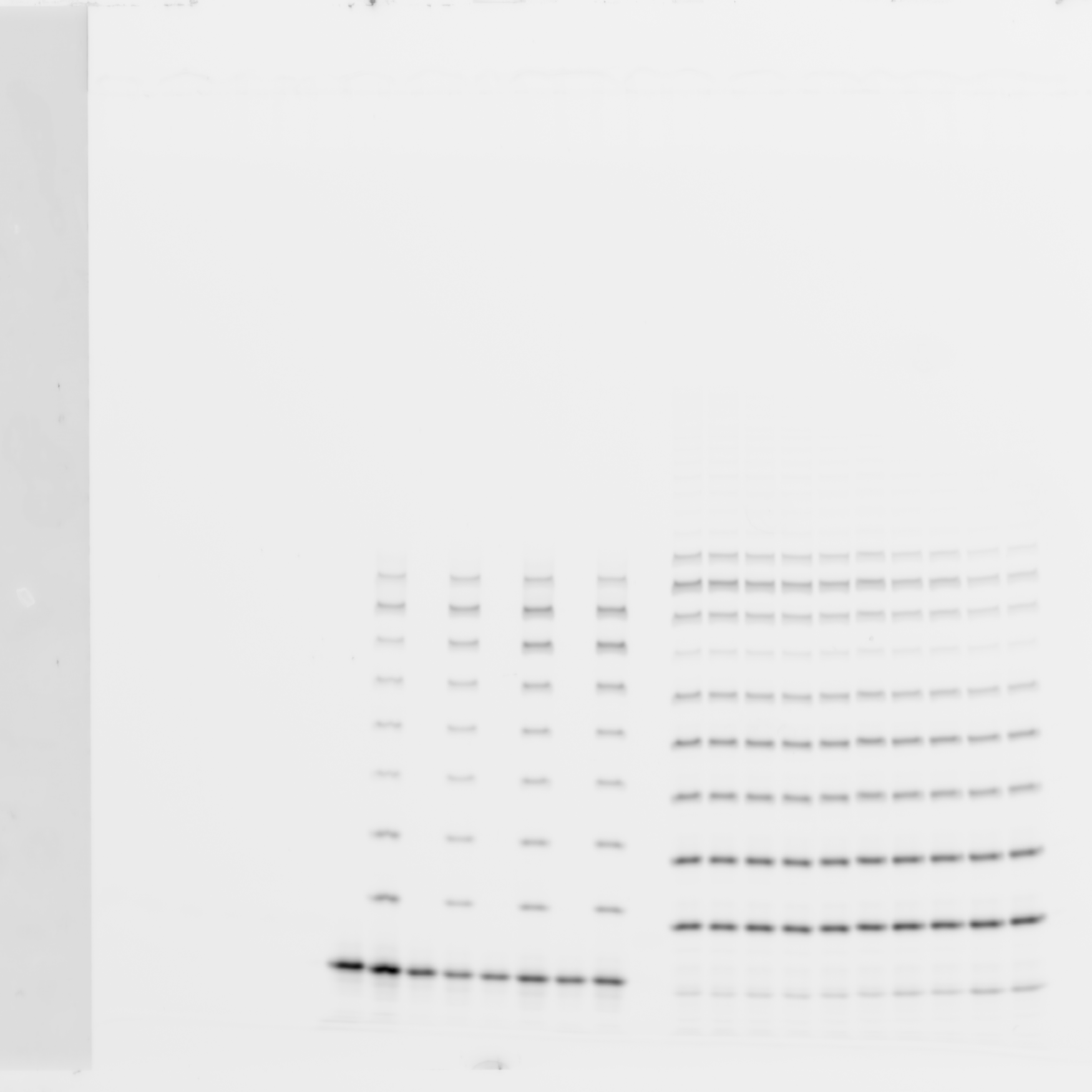

Supplement: Figure 2—source data 1. [file elife-75186-fig2-data1.zip › Figure 2-source data 1/Original gel files/20191001-173612-FITC.gel]

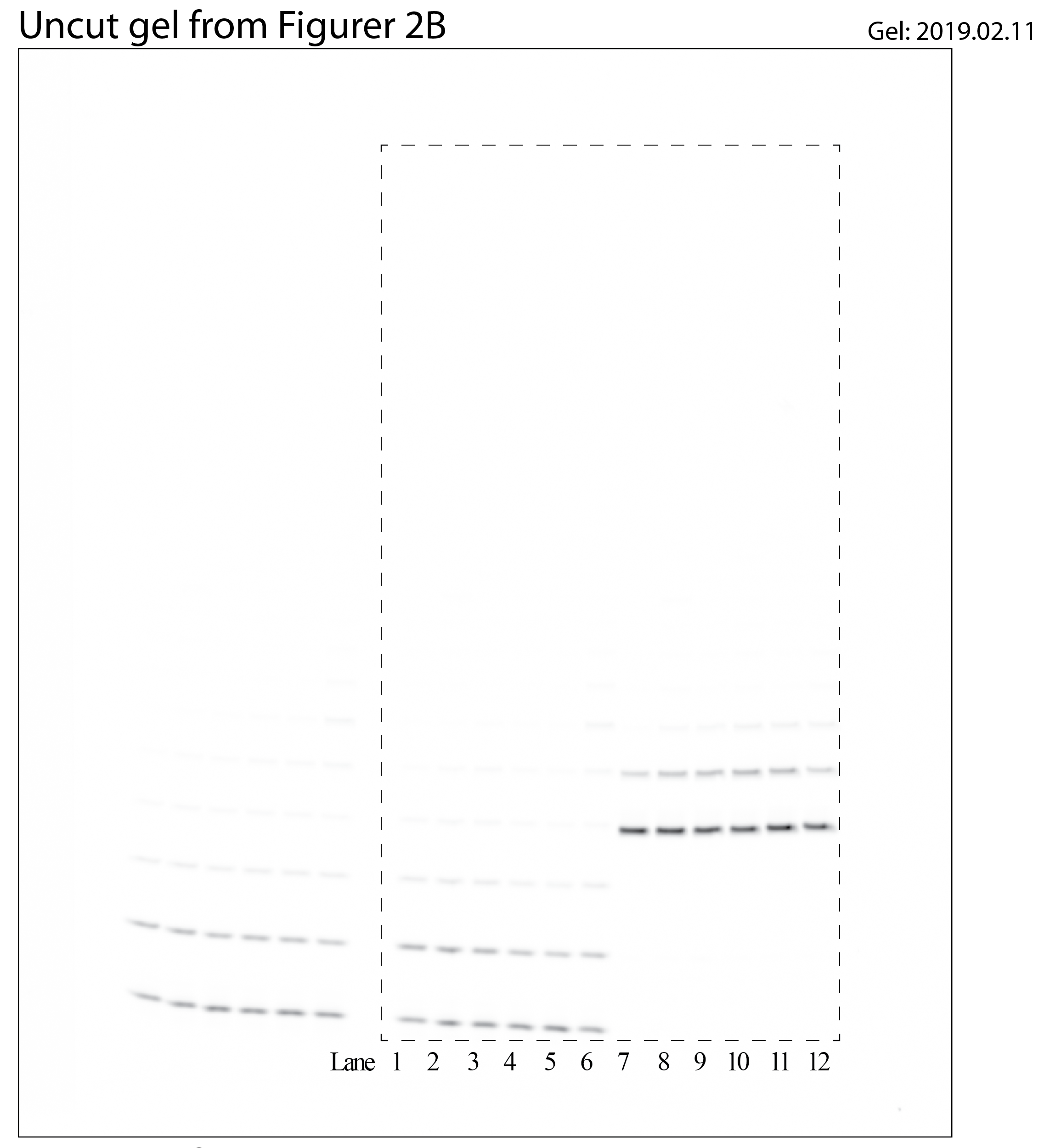

Supplement: Figure 2—source data 1. [file elife-75186-fig2-data1.zip › Figure 2-source data 1/Uncut gel from Figure 2B.png]

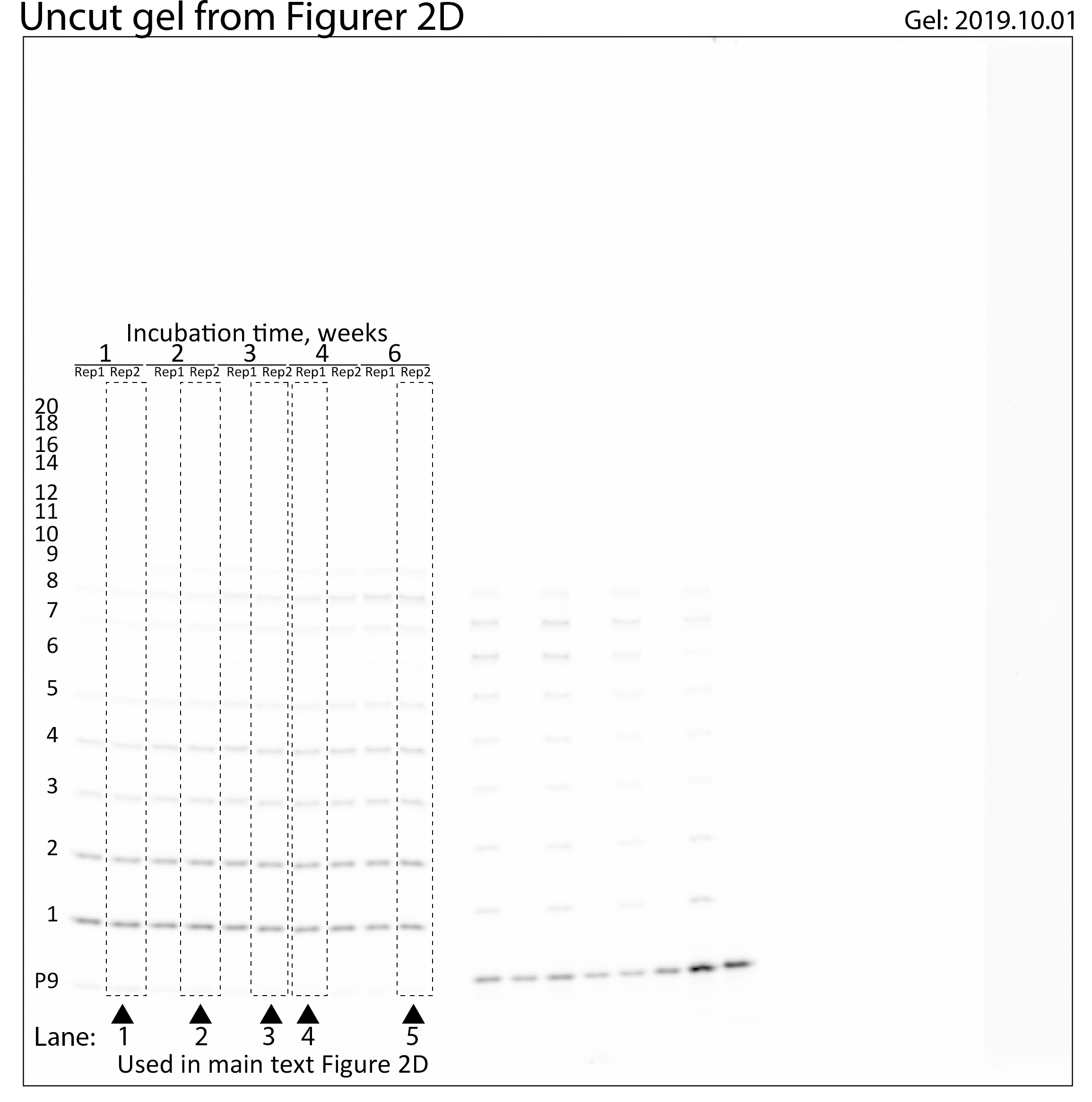

Supplement: Figure 2—source data 1. [file elife-75186-fig2-data1.zip › Figure 2-source data 1/Uncut gel from Figure 2D.png]

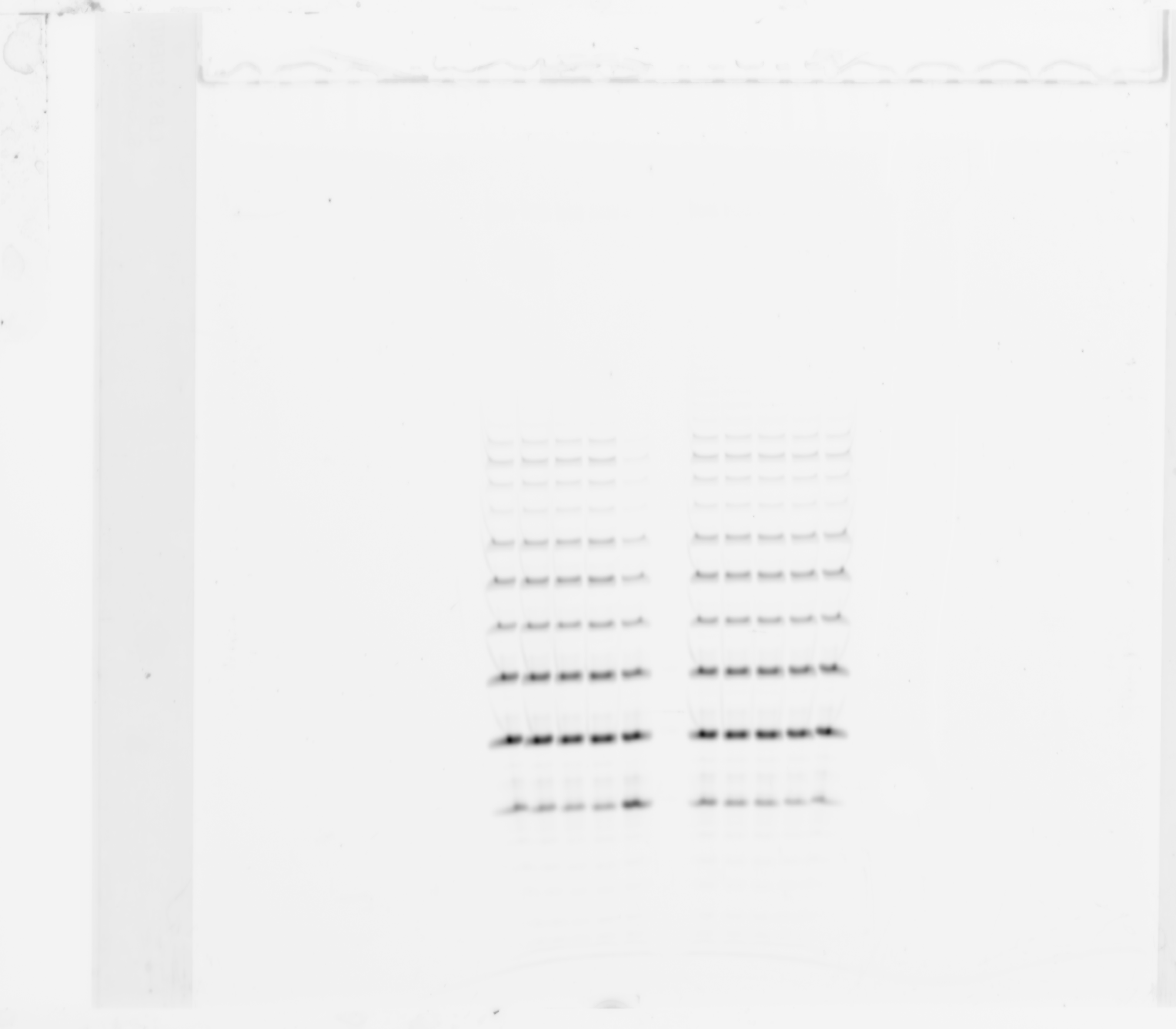

Supplement: Figure 2—figure supplement 1—source data 1. [file elife-75186-fig2-figsupp1-data1.zip › Figure 2-Figure supplement 1-source data 1/original gel files/20190605_180522-FITC.gel]

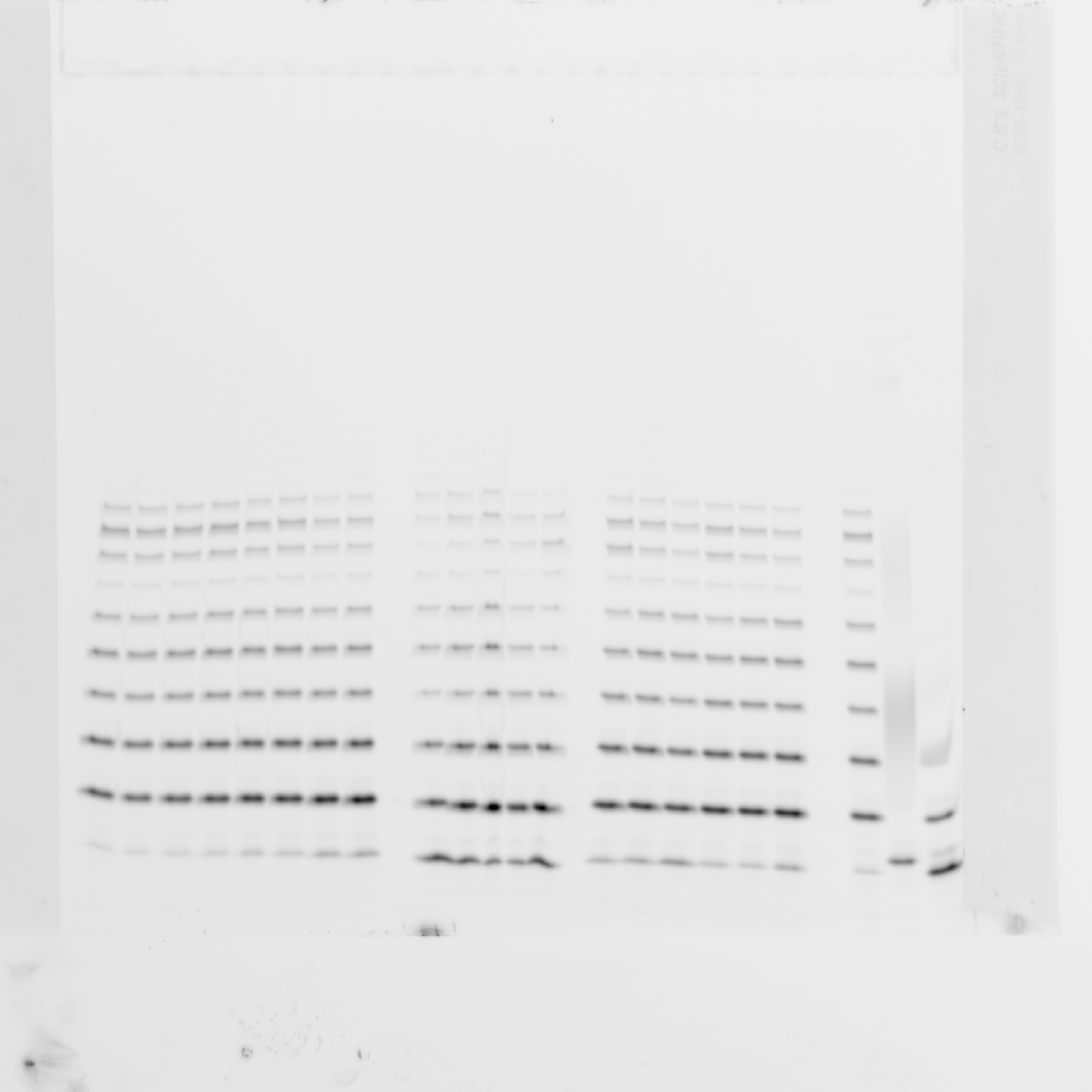

Supplement: Figure 2—figure supplement 1—source data 1. [file elife-75186-fig2-figsupp1-data1.zip › Figure 2-Figure supplement 1-source data 1/original gel files/20190912-155231-FITC.gel]

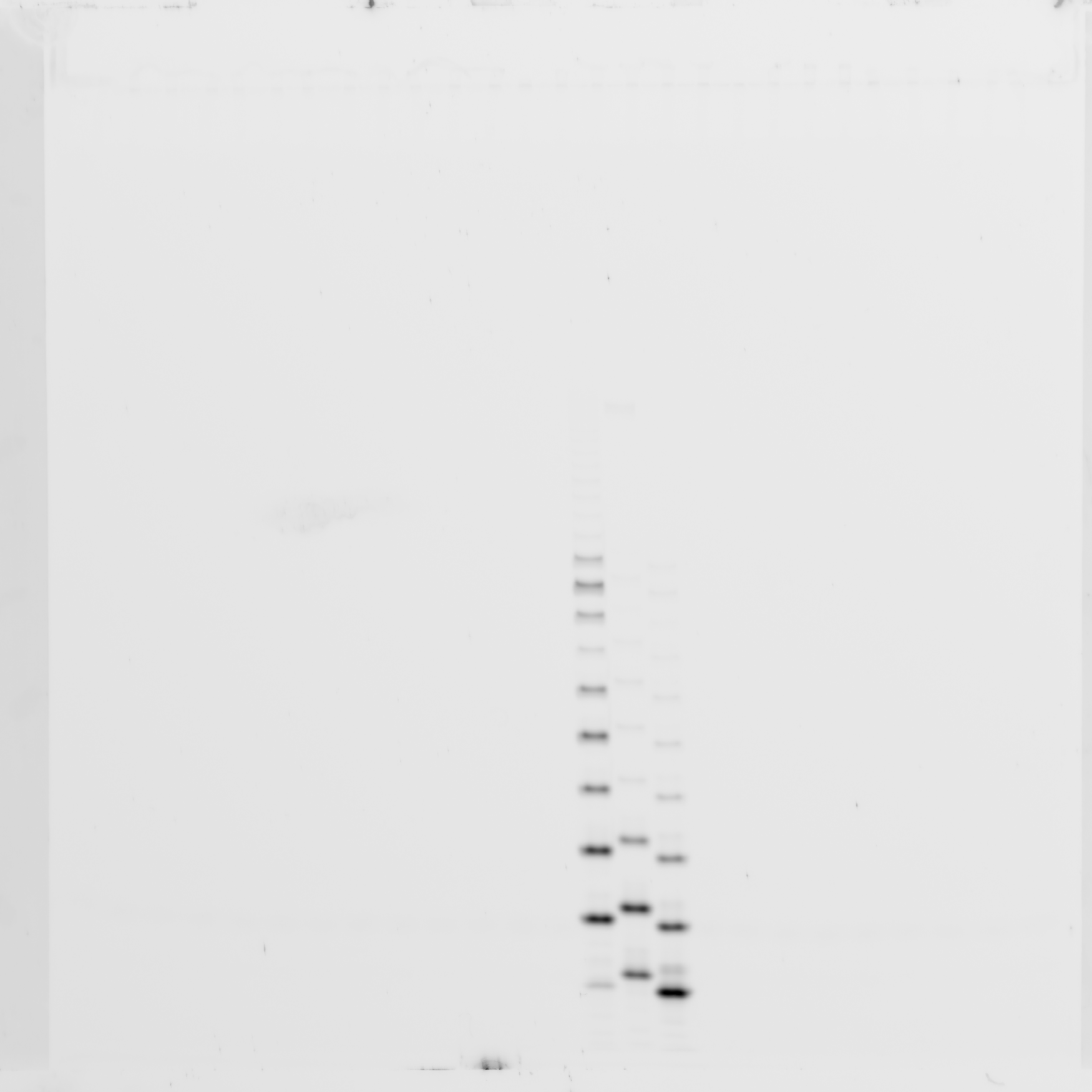

Supplement: Figure 2—figure supplement 1—source data 1. [file elife-75186-fig2-figsupp1-data1.zip › Figure 2-Figure supplement 1-source data 1/original gel files/20190916-153845-FITC.gel]

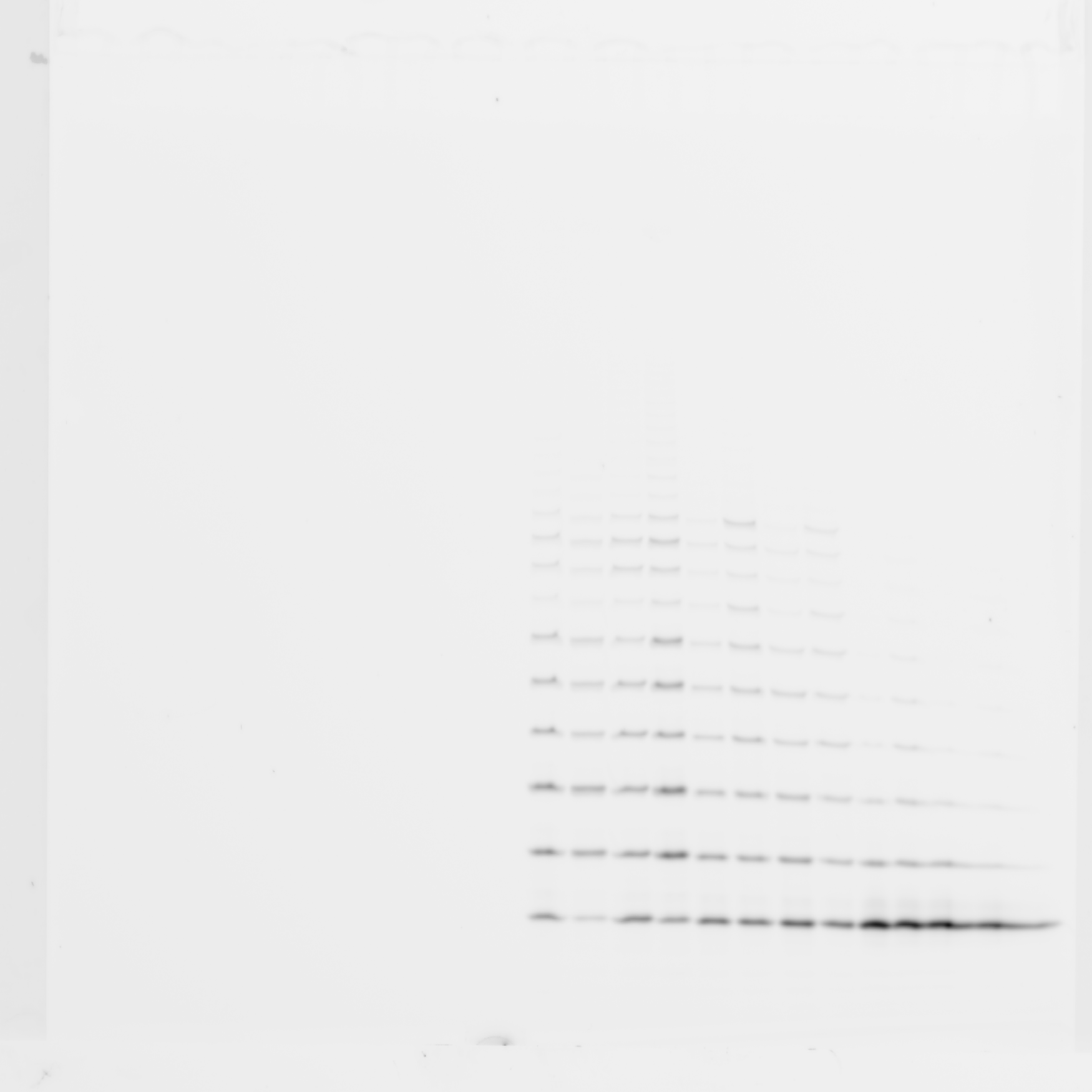

Supplement: Figure 2—figure supplement 1—source data 1. [file elife-75186-fig2-figsupp1-data1.zip › Figure 2-Figure supplement 1-source data 1/original gel files/20200106-145855-FITC.gel]

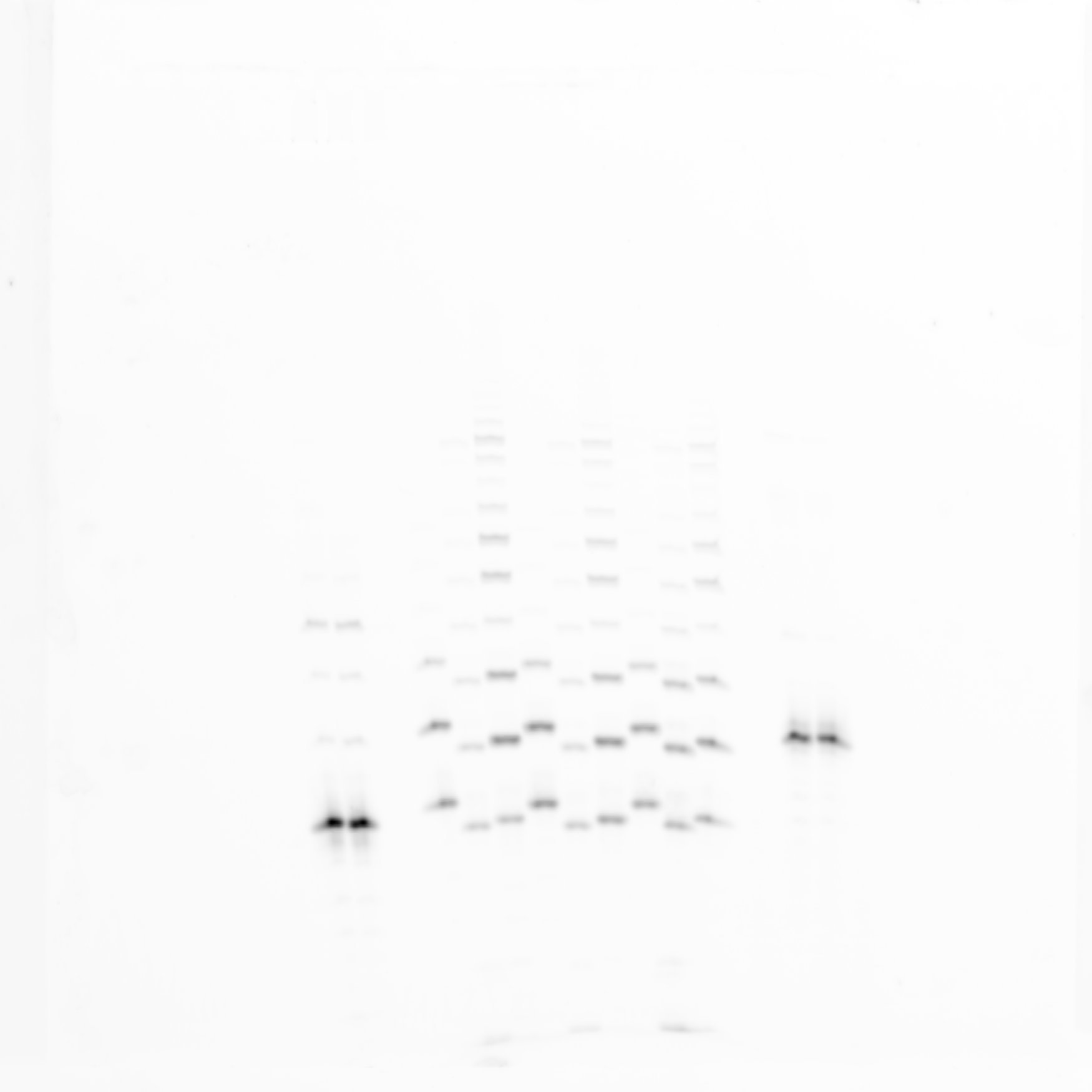

Supplement: Figure 2—figure supplement 1—source data 1. [file elife-75186-fig2-figsupp1-data1.zip › Figure 2-Figure supplement 1-source data 1/original gel files/20201102-175314-FITC.gel]

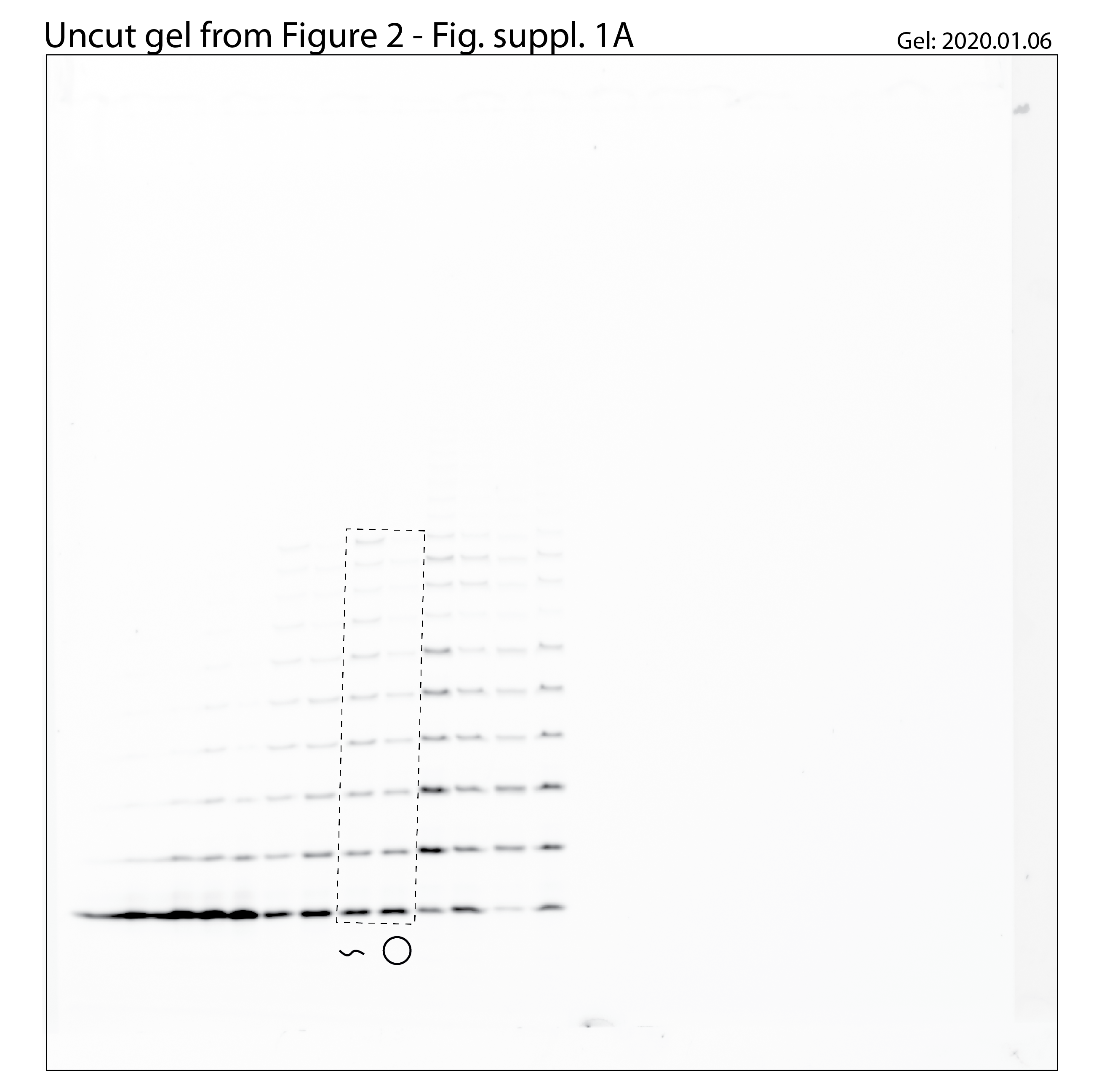

Supplement: Figure 2—figure supplement 1—source data 1. [file elife-75186-fig2-figsupp1-data1.zip › Figure 2-Figure supplement 1-source data 1/Uncut gel from Figure 2 - Fig. suppl. 1A (Gel 2020.01.06).png]

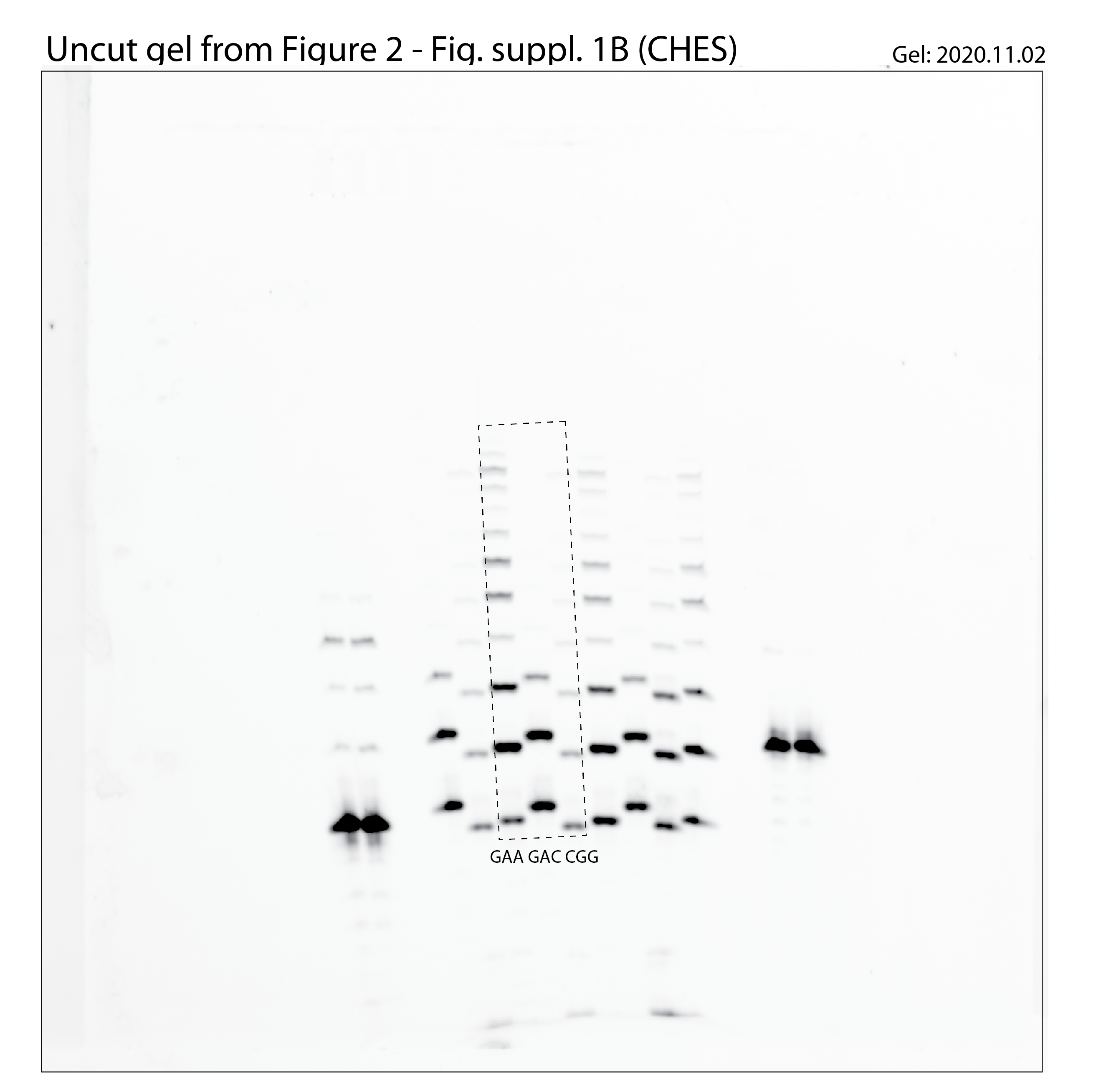

Supplement: Figure 2—figure supplement 1—source data 1. [file elife-75186-fig2-figsupp1-data1.zip › Figure 2-Figure supplement 1-source data 1/Uncut gel from Figure 2 - Fig. suppl. 1B (CHES) (Gel 2020.11.02).png]

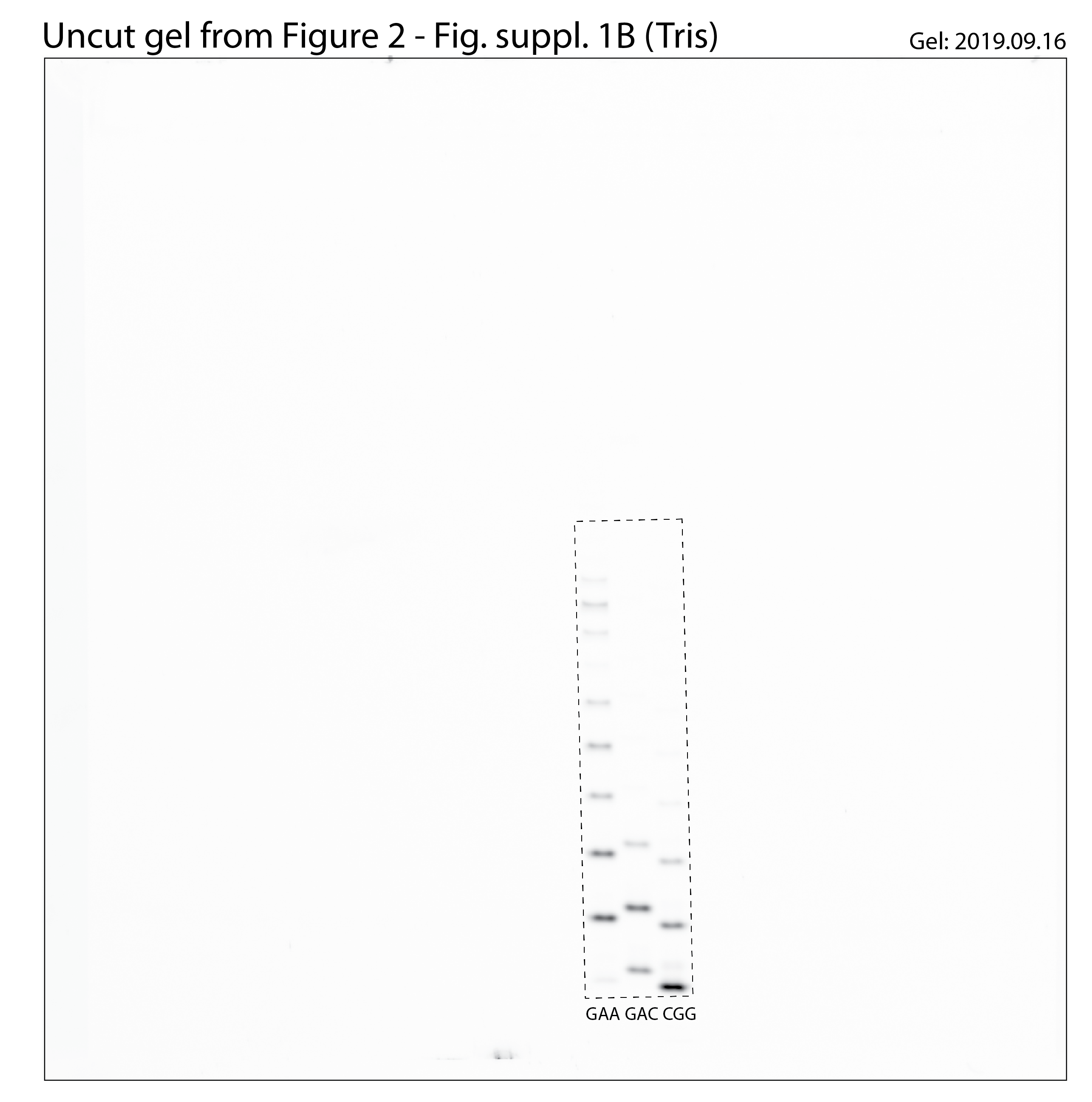

Supplement: Figure 2—figure supplement 1—source data 1. [file elife-75186-fig2-figsupp1-data1.zip › Figure 2-Figure supplement 1-source data 1/Uncut gel from Figure 2 - Fig. suppl. 1B (Tris) (Gel 2019.09.16).png]

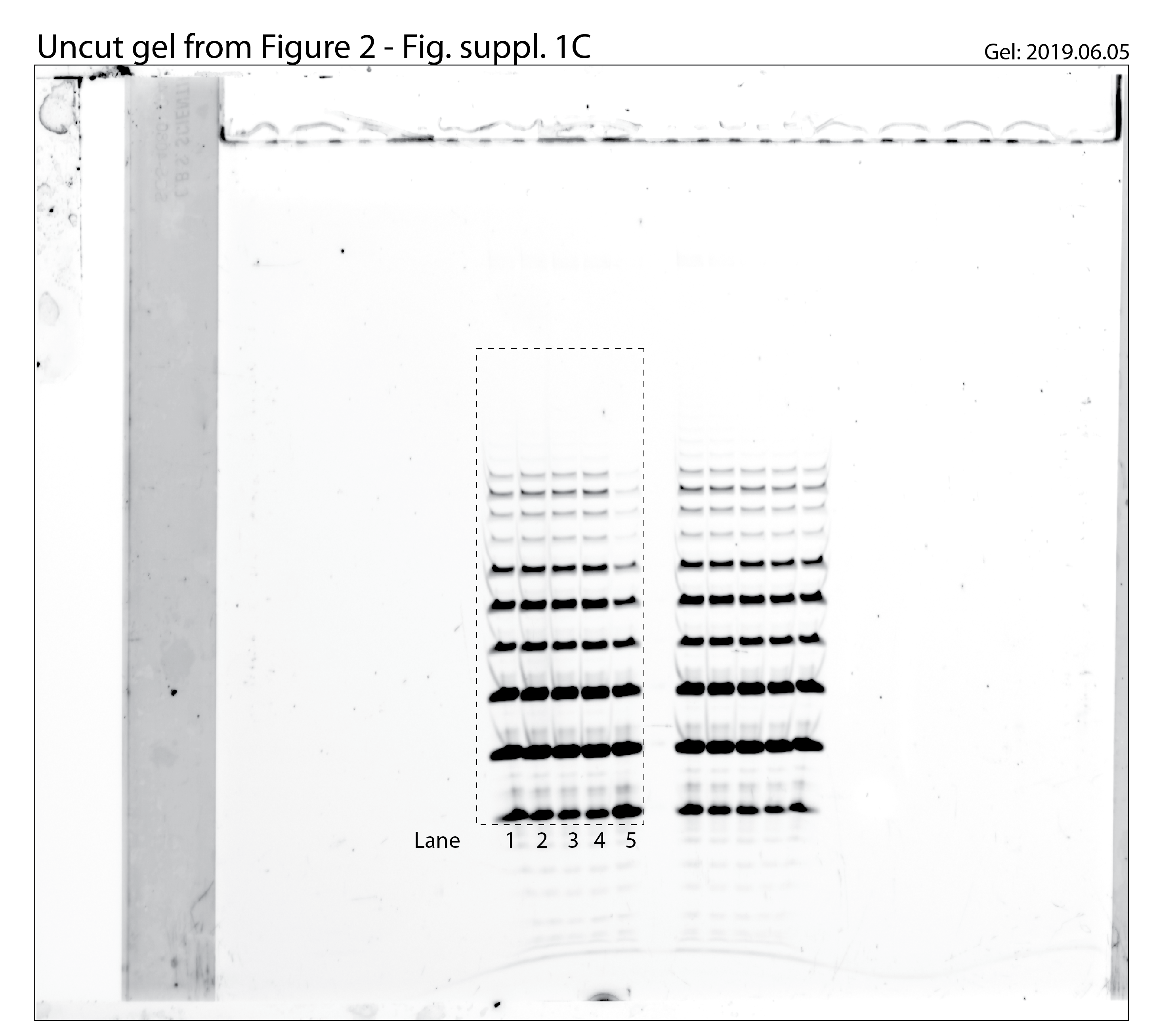

Supplement: Figure 2—figure supplement 1—source data 1. [file elife-75186-fig2-figsupp1-data1.zip › Figure 2-Figure supplement 1-source data 1/Uncut gel from Figure 2 - Fig. suppl. 1C (Gel 2019.06.05).png]

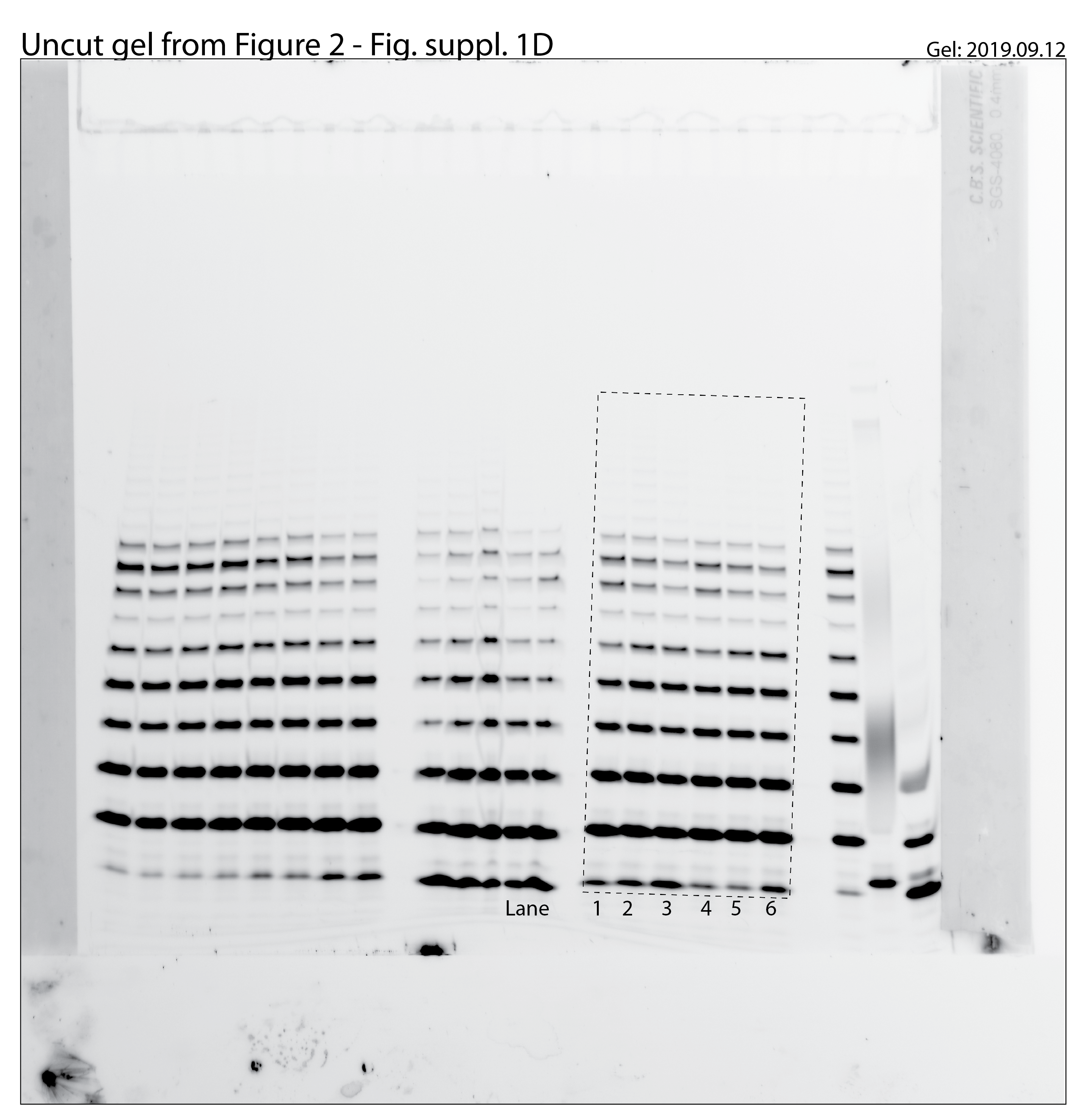

Supplement: Figure 2—figure supplement 1—source data 1. [file elife-75186-fig2-figsupp1-data1.zip › Figure 2-Figure supplement 1-source data 1/Uncut gel from Figure 2 - Fig. suppl. 1D (Gel 2019.09.12).png]

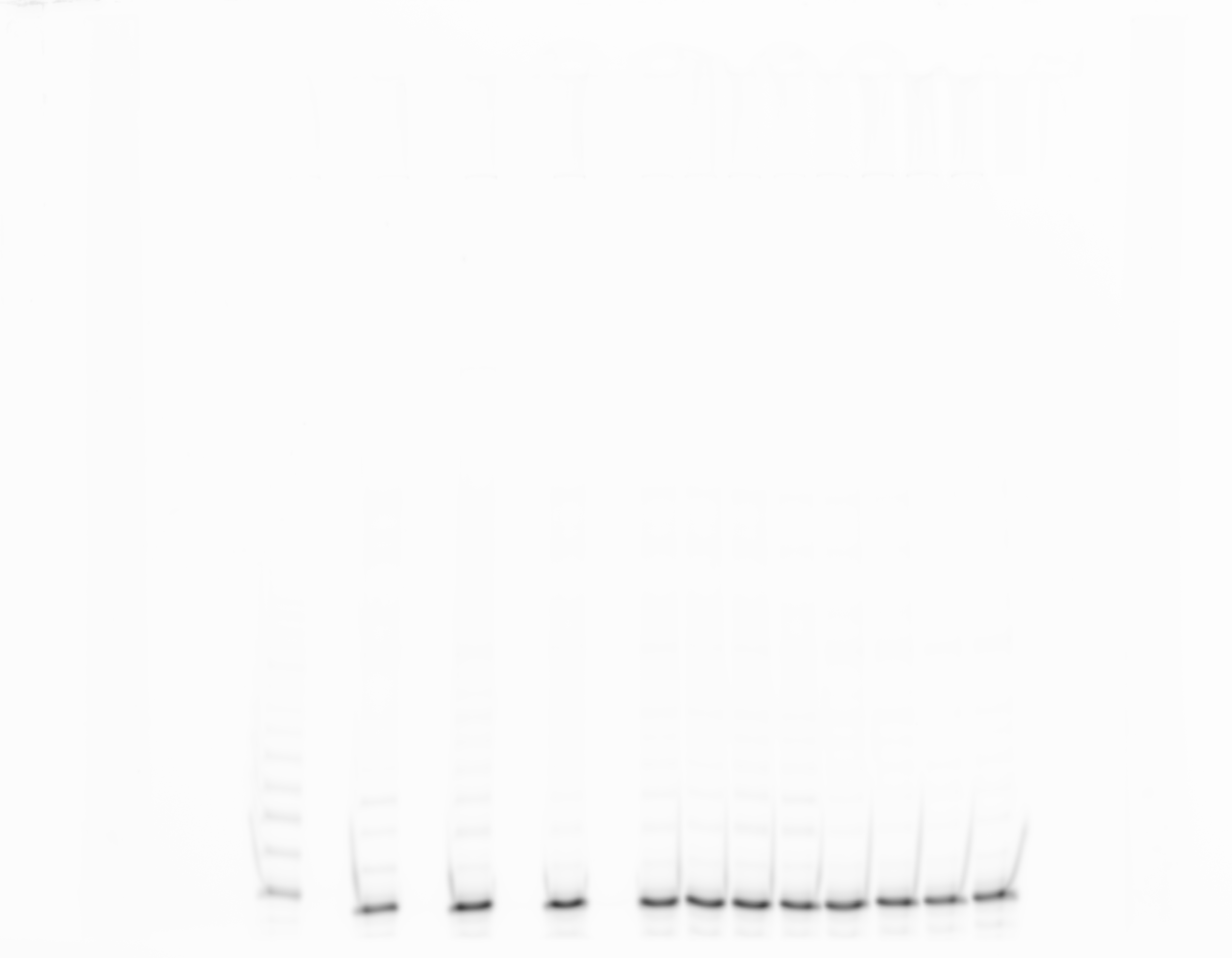

Supplement: Figure 4—figure supplement 1—source data 1. [file elife-75186-fig4-figsupp1-data1.zip › Figure 4-Figure supplement 1-source data 1/Original gel files/20200819-155123-FITC.gel]

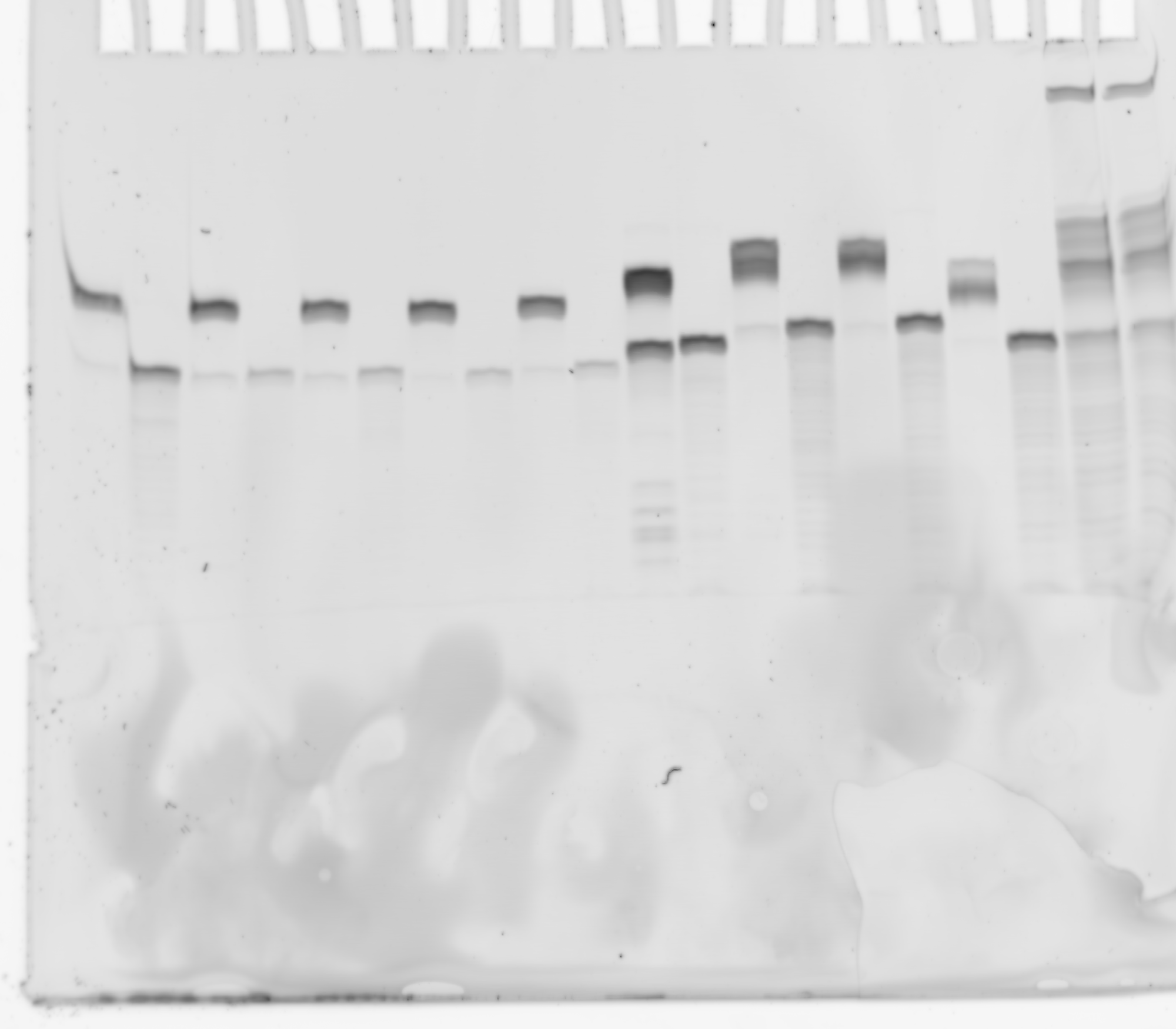

Supplement: Figure 4—figure supplement 1—source data 1. [file elife-75186-fig4-figsupp1-data1.zip › Figure 4-Figure supplement 1-source data 1/Original gel files/20201029-155836-[Cy2].gel]

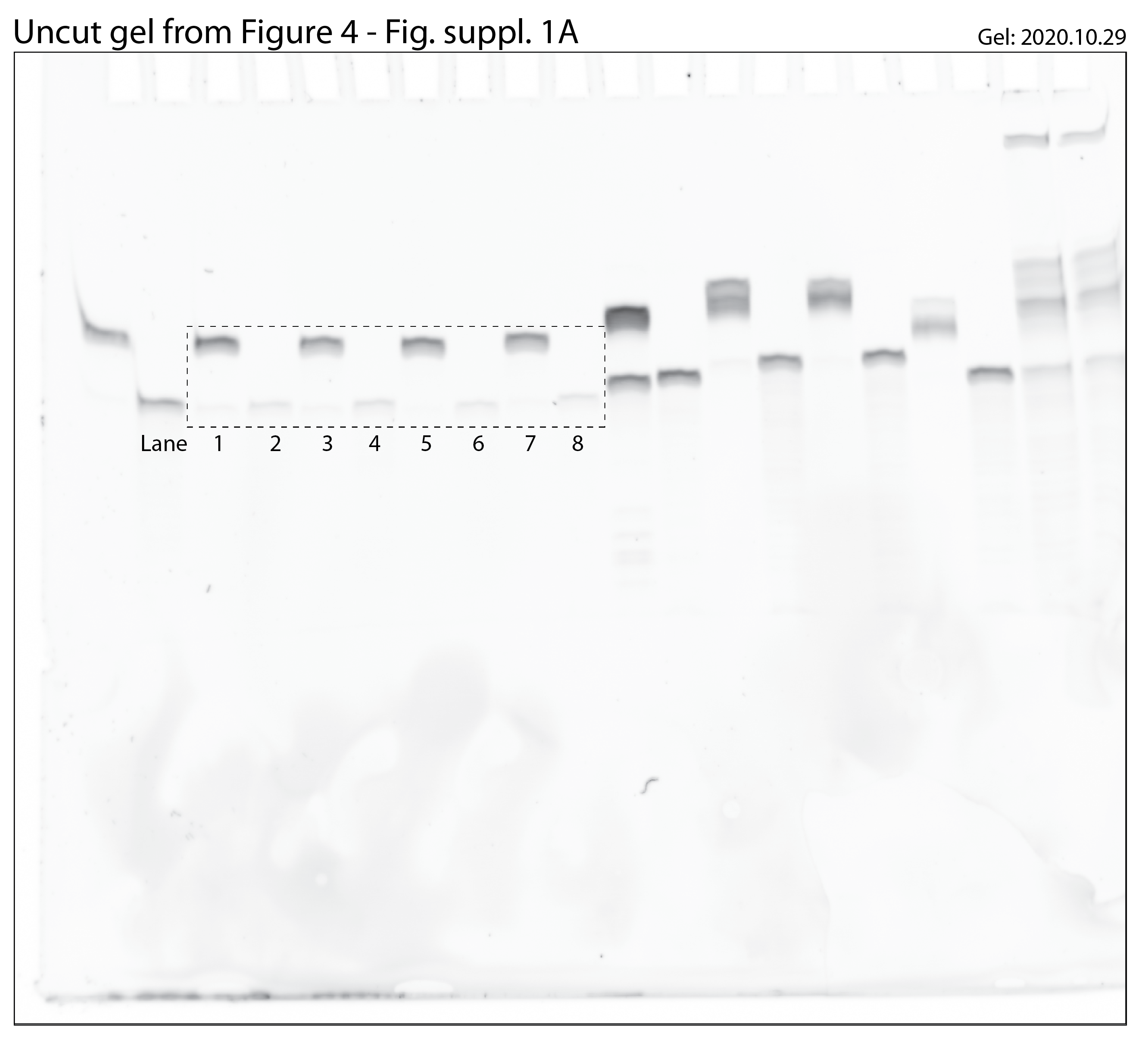

Supplement: Figure 4—figure supplement 1—source data 1. [file elife-75186-fig4-figsupp1-data1.zip › Figure 4-Figure supplement 1-source data 1/Uncut gel from Figure 4 - Fig. suppl. 1A (Gel 2020.10.29).png]

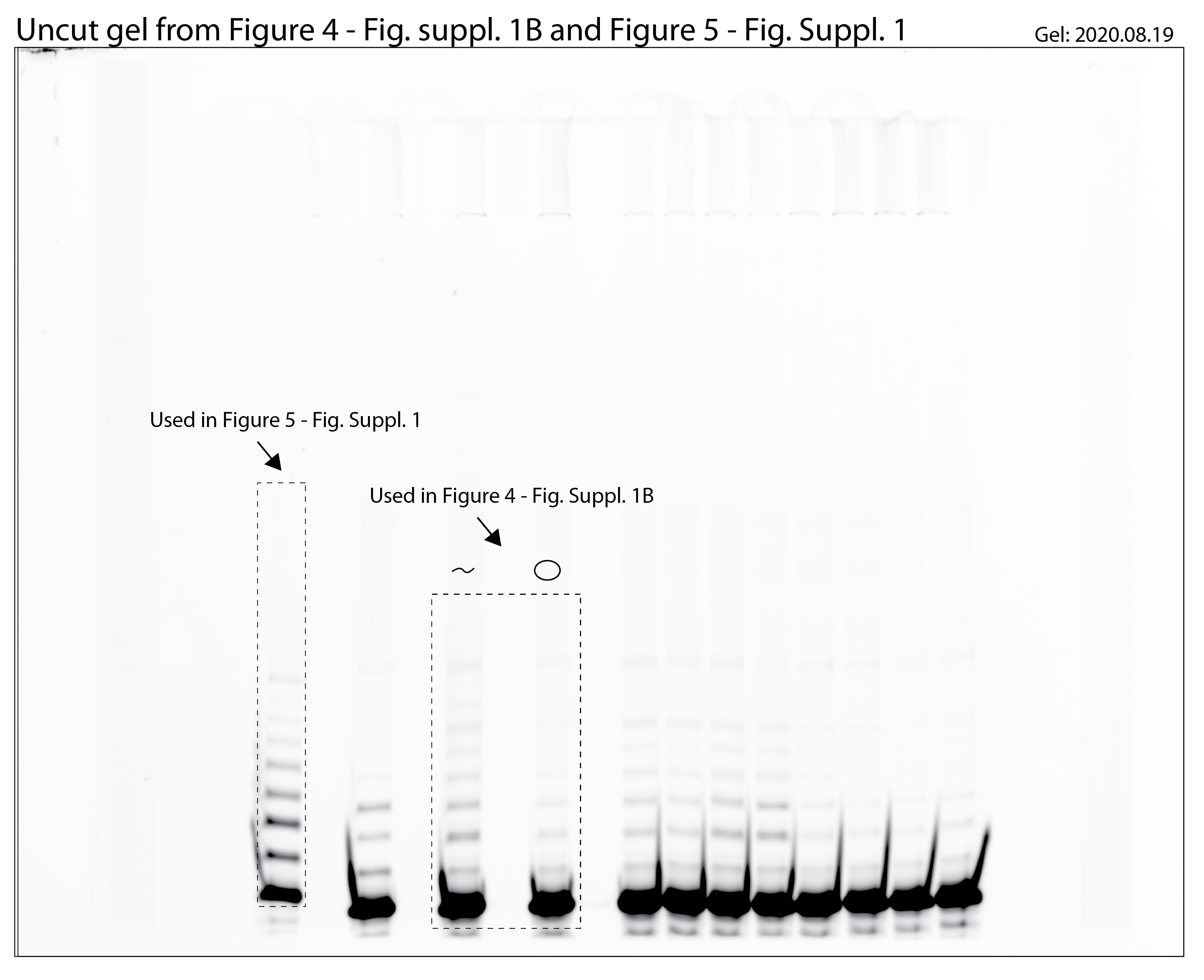

Supplement: Figure 4—figure supplement 1—source data 1. [file elife-75186-fig4-figsupp1-data1.zip › Figure 4-Figure supplement 1-source data 1/Uncut gel from Figure 4 - Fig. suppl. 1B (Gel 2020.08.19).png]

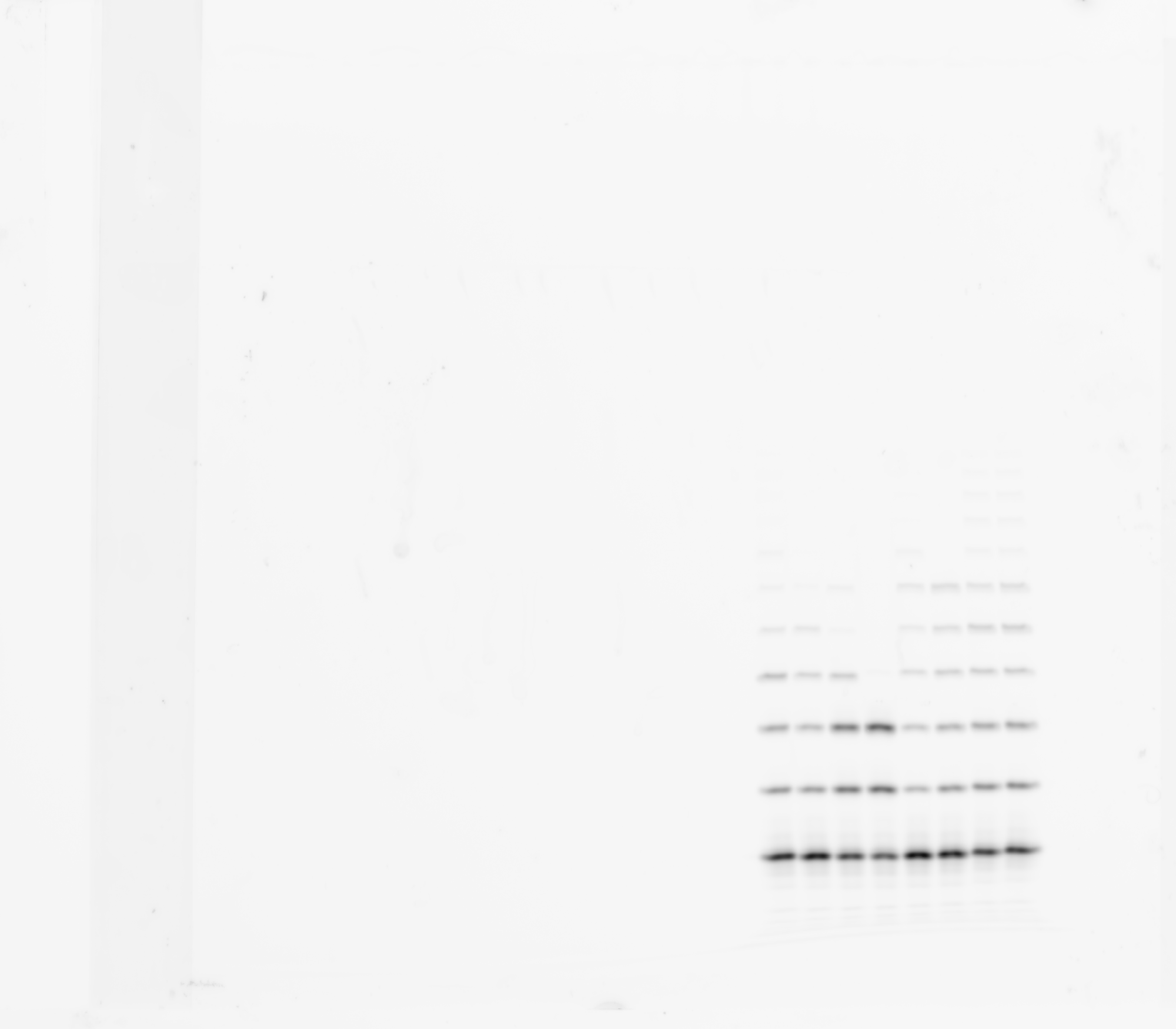

Supplement: Figure 5—source data 1. [file elife-75186-fig5-data1.zip › Figure 5-source data 1/Original gel files/20200115-175412-FITC.gel]

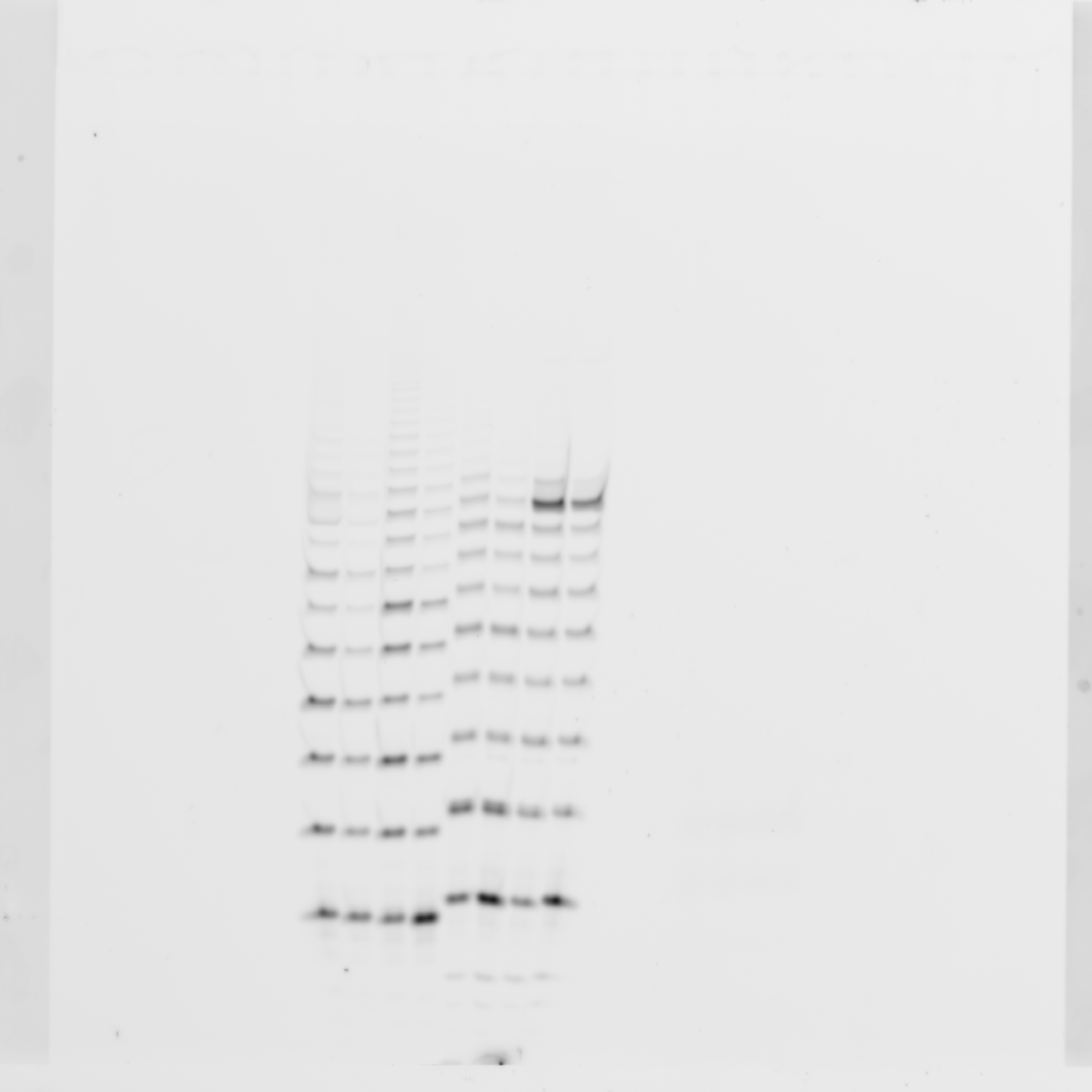

Supplement: Figure 5—source data 1. [file elife-75186-fig5-data1.zip › Figure 5-source data 1/Original gel files/20200131-164751-FITC.gel]

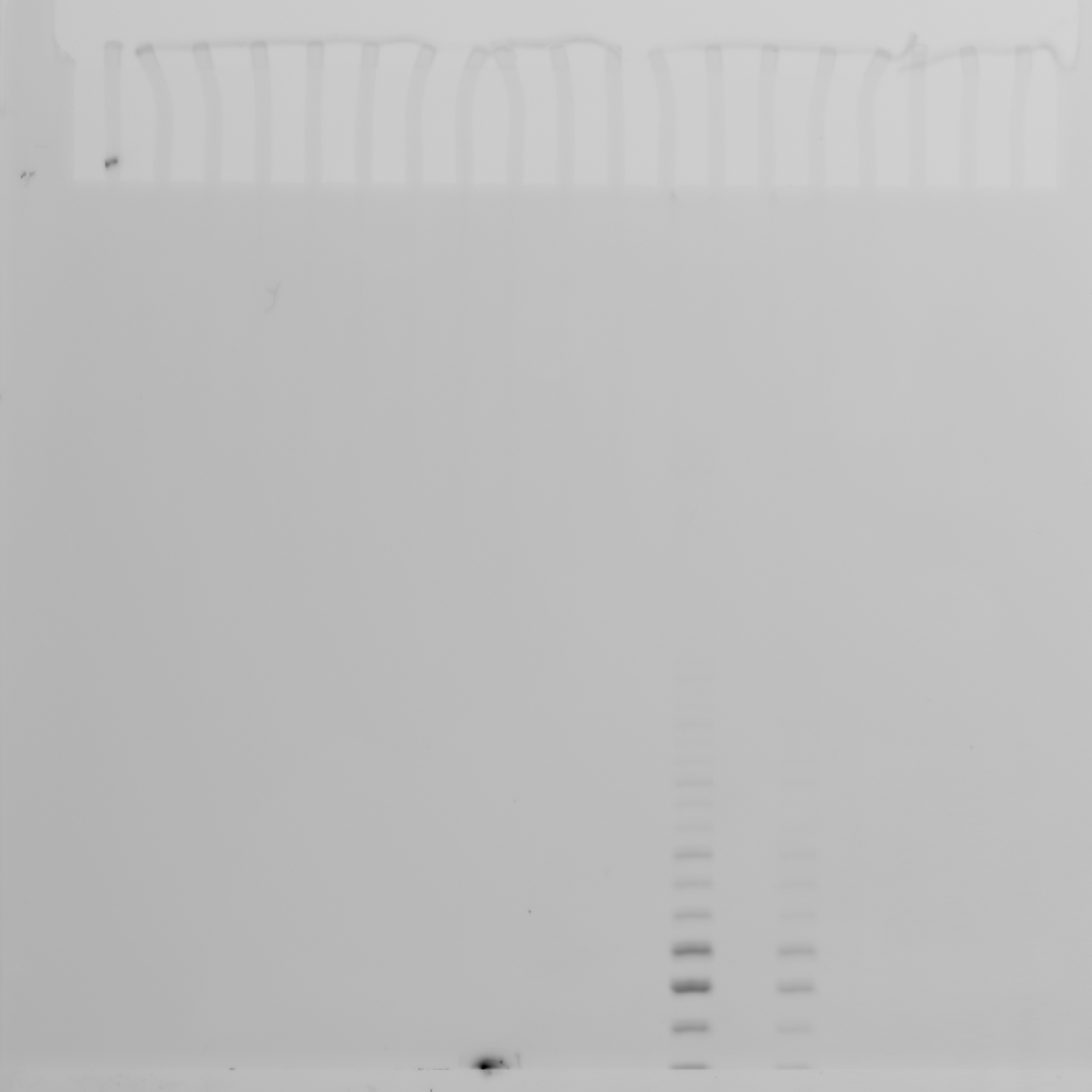

Supplement: Figure 5—source data 1. [file elife-75186-fig5-data1.zip › Figure 5-source data 1/Original gel files/20201215-141325-FITC.gel]

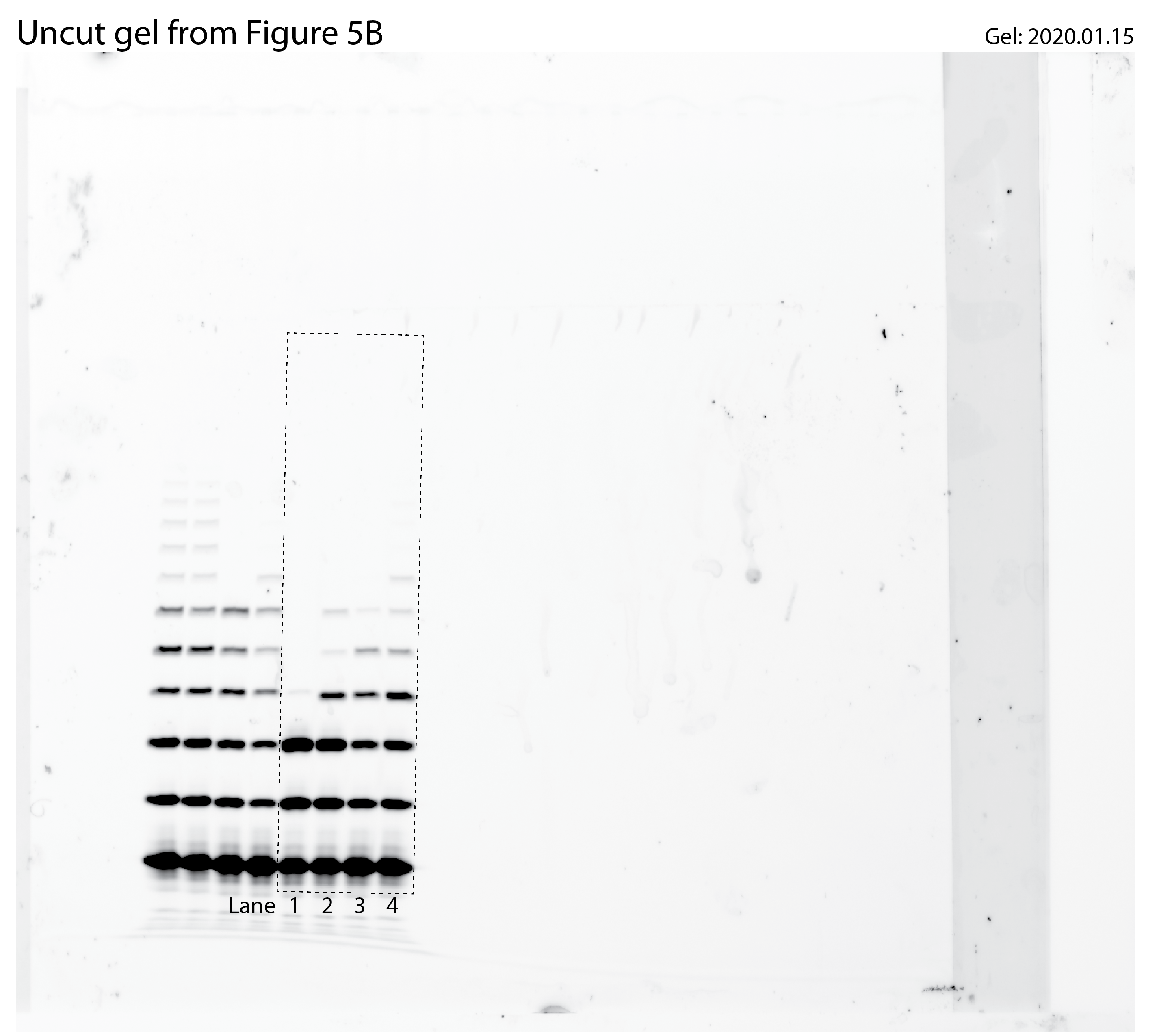

Supplement: Figure 5—source data 1. [file elife-75186-fig5-data1.zip › Figure 5-source data 1/Uncut gel from Figure 5B, Lane1to4.png]

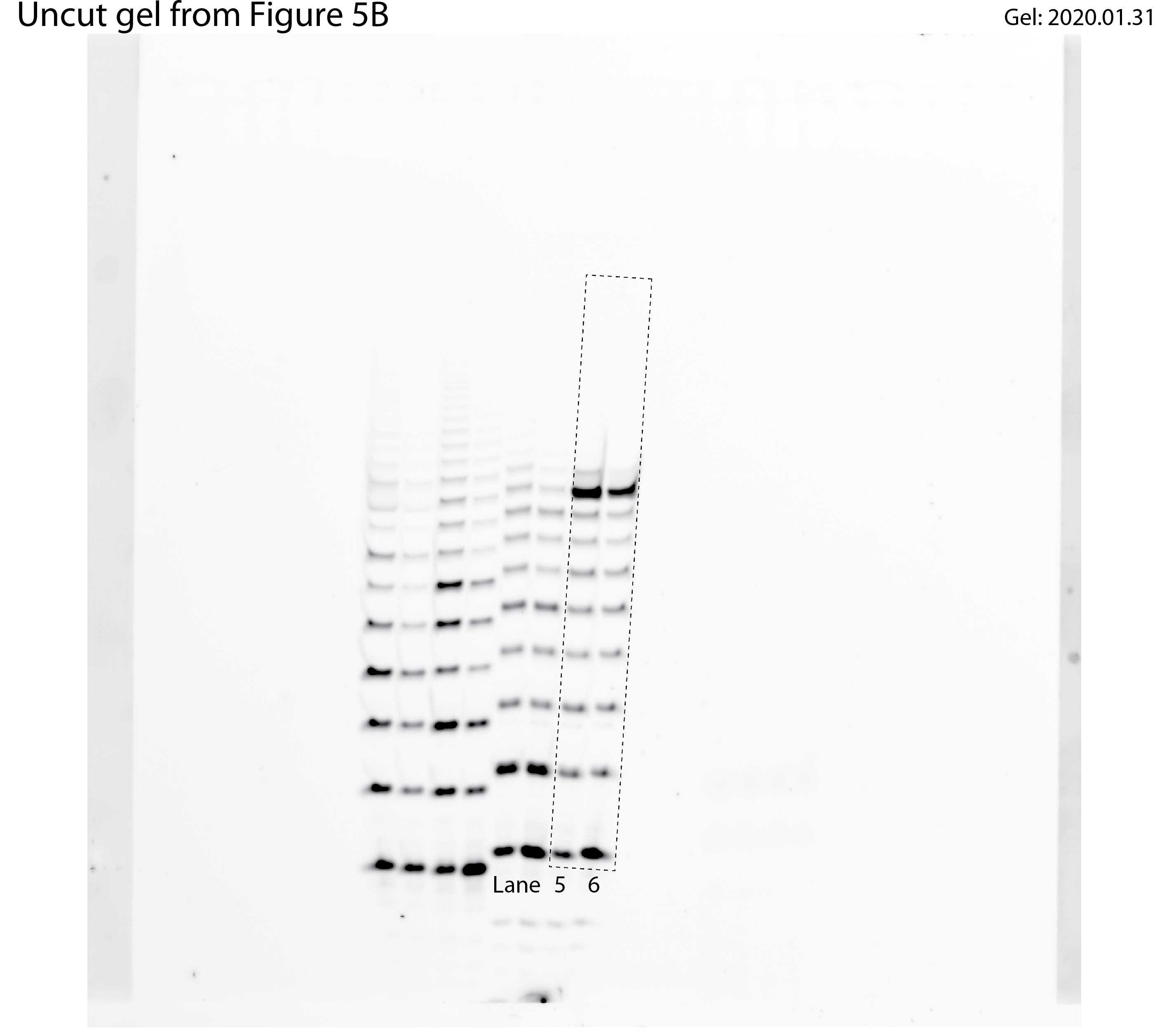

Supplement: Figure 5—source data 1. [file elife-75186-fig5-data1.zip › Figure 5-source data 1/Uncut gel from Figure 5B, Lane5,6.png]

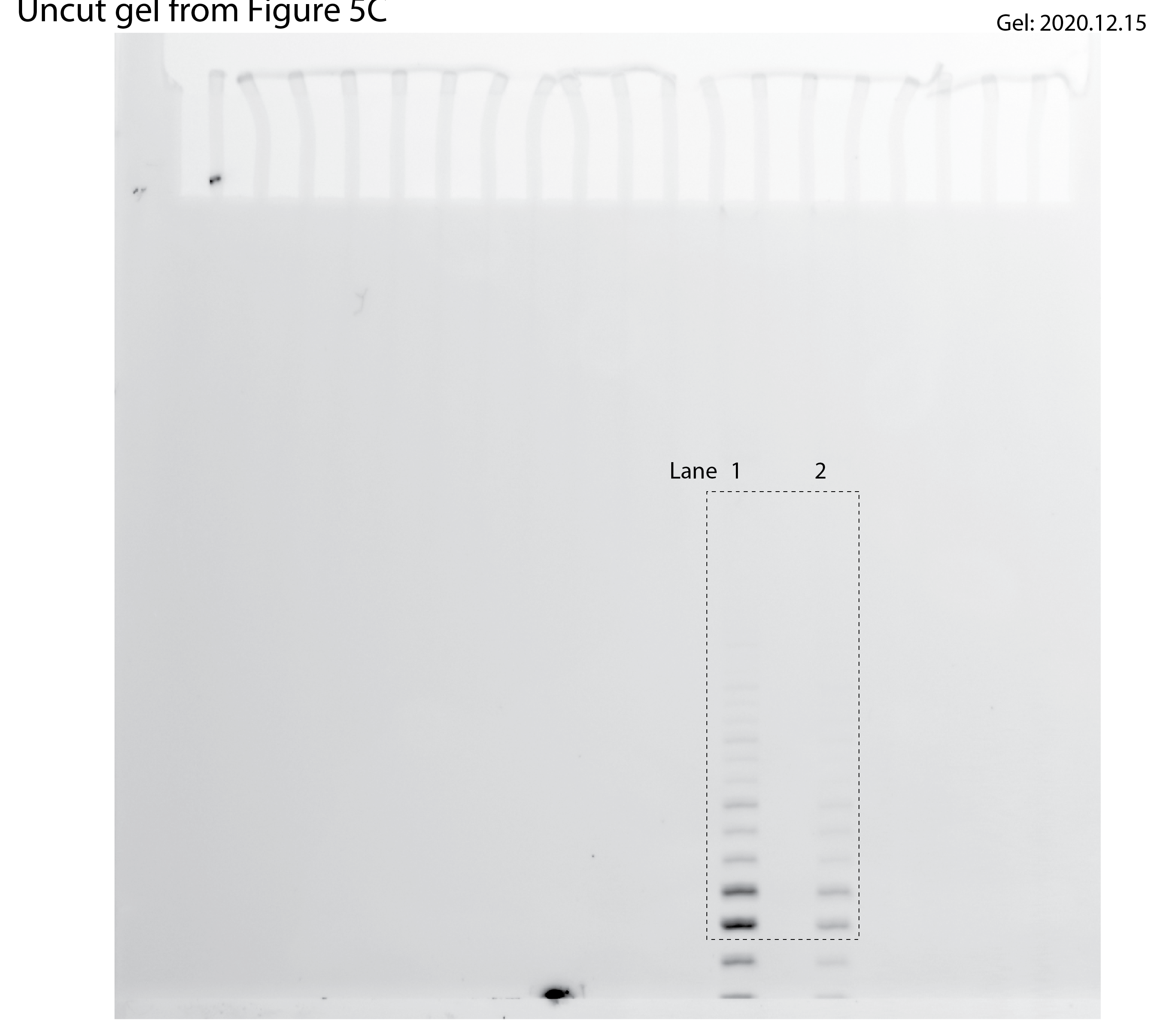

Supplement: Figure 5—source data 1. [file elife-75186-fig5-data1.zip › Figure 5-source data 1/Uncut gel from Figure 5C.png]

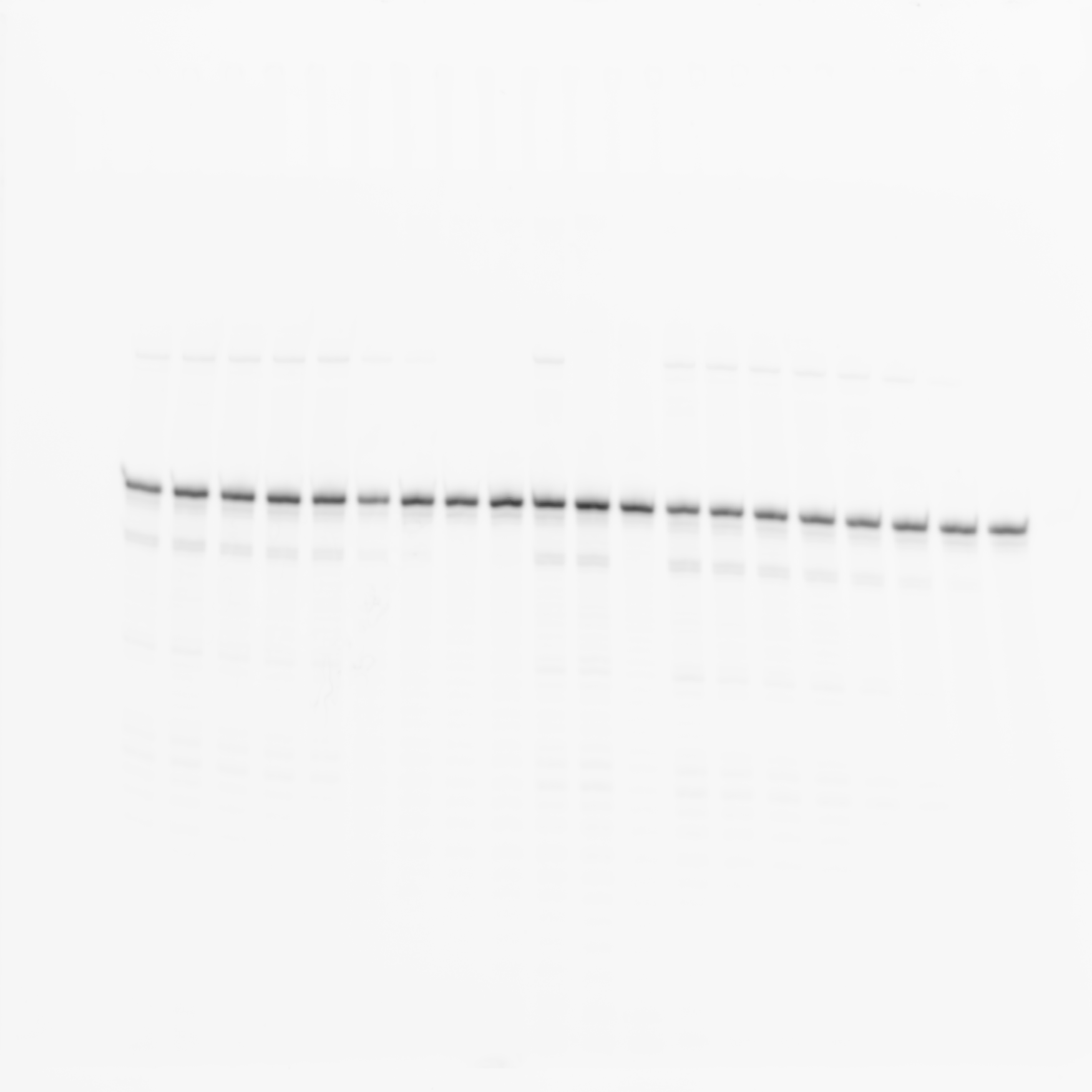

Supplement: Figure 6—source data 1. [file elife-75186-fig6-data1.zip › Figure 6-source data 1/Original gel files/20201124-172454-FITC.gel]

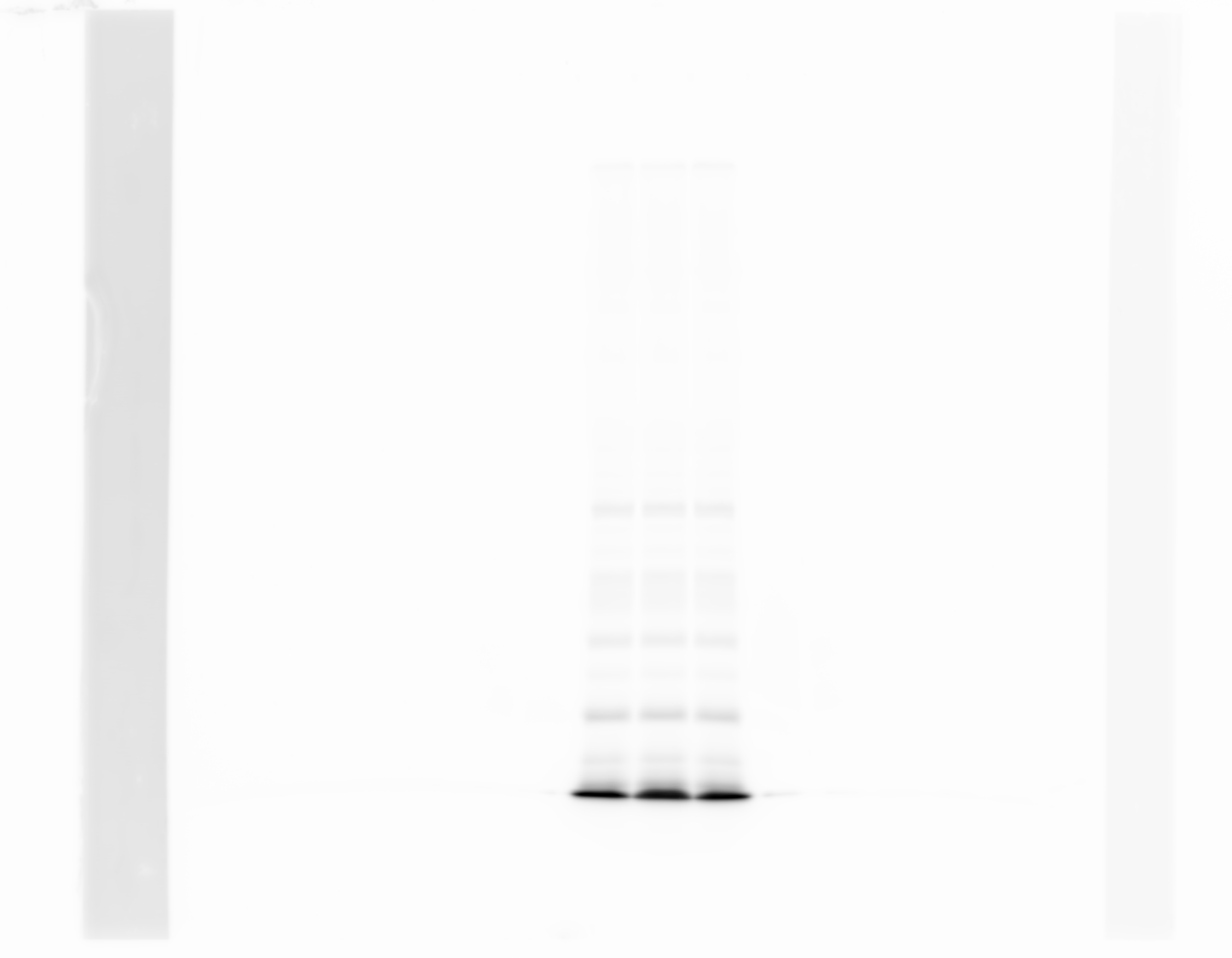

Supplement: Figure 6—source data 1. [file elife-75186-fig6-data1.zip › Figure 6-source data 1/Original gel files/20201207-174524-FITC.gel]

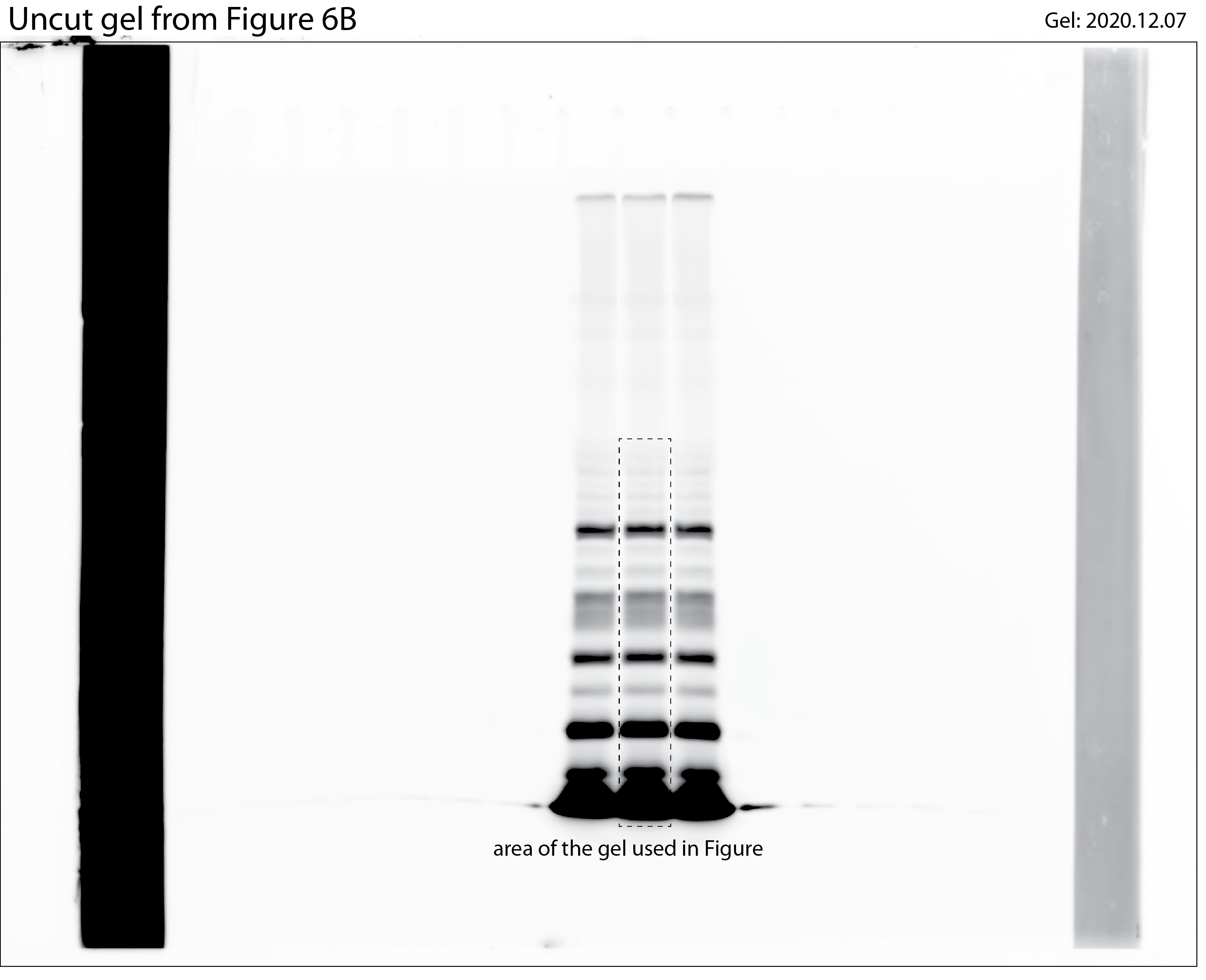

Supplement: Figure 6—source data 1. [file elife-75186-fig6-data1.zip › Figure 6-source data 1/Uncut gel from Figure 6B.png]

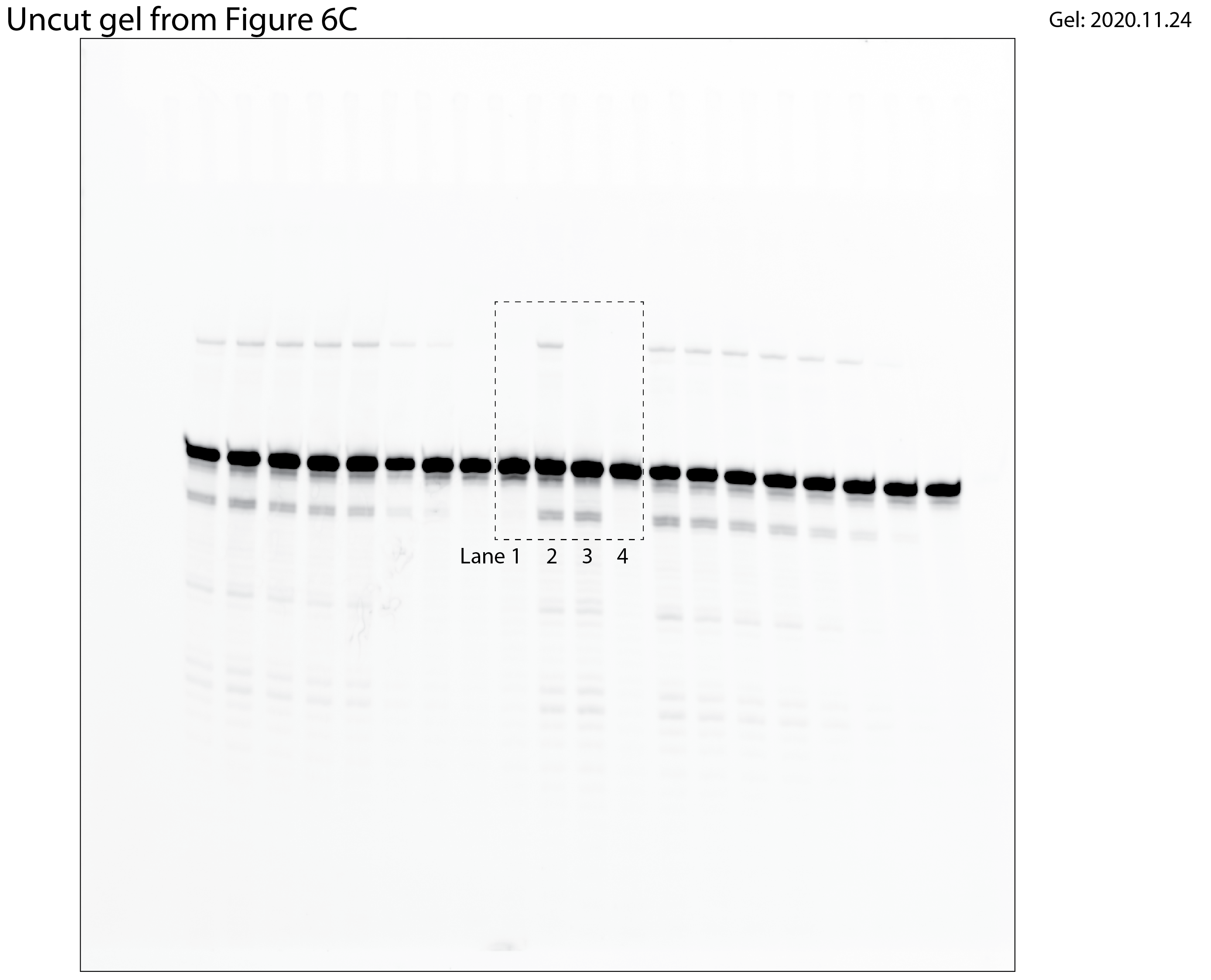

Supplement: Figure 6—source data 1. [file elife-75186-fig6-data1.zip › Figure 6-source data 1/Uncut gel from Figure 6C.png]
