# Supplementary material for: Rolling circle RNA synthesis catalyzed by RNA
Source: eLife. 2022 Feb 2;11:e75186. doi: 10.7554/eLife.75186 (PMC8937235; doi:10.7554/eLife.75186)
Supplement: Supplementary file 1. [file elife-75186-supp1.docx]

## **Supplementary file 1**

## Oligonucleotide sequences

| ***Oligo name*** | ***Seq. (5' to 3')*** | ***type*** | ***Notes:*** |
| --- | --- | --- | --- |
| **Templates** |  |  |  |
| 12GAA-p  (termed sc12GAA-p when circularized) | /5Phos/UUC UUC UUC UUC UUC UUC UUC UUC UUC UUC UUC UUC | RNA | /5Phos/ indicated that the RNA is 5’-phosphorylated |
| sc12GAC-p | GATCGATCTCGCCCGCGAAATTAATACGACTCACTATA-GTCGTCGTCGTCGTCGTCGTCGTCGTCGTCGTCGTC-GGGTCGGCATGGCATC | DNA | Fill-in with HDVrt (as back (Ba) primer) and *in vitro* transcribe |
| sc12CGG-p | GATCGATCTCGCCCGCGAAATTAATACGACTCACTATA-GGCGGCGGCGGCGGCGGCGGCGGCGGCGGCGGCGGC-GGGTCGGCATGGCATC | DNA | Fill-in with HDVrt (as Ba primer) and *in vitro* transcribe |
| scGAA8 | GATCGATCTCGCCCGCGAAATTAATACGACTCACTATAGGCAGTTCTTCTTCTTCTTCTTCTTCTTCCGGTTGGGTCGGCATGGCATC | DNA | Fill-in with HDVrt (as Ba primer) and *in vitro* transcribe |
| scGAA9 | GATCGATCTCGCCCGCGAAATTAATACGACTCACTATAGGCAGTTCTTCTTCTTCTTCTTCTTCTTCTTCCGGTTGGGTCGGCATGGCATC | DNA | Fill-in with HDVrt (as Ba primer) and *in vitro* transcribe |
| scGAA10 | GATCGATCTCGCCCGCGAAATTAATACGACTCACTATAGGCAGTTCTTCTTCTTCTTCTTCTTCTTCTTCTTCCGGTTGGGTCGGCATGGCATC | DNA | Fill-in with HDVrt (as Ba primer) and *in vitro* transcribe |
| scGAA11 | GATCGATCTCGCCCGCGAAATTAATACGACTCACTATAGGCAGTTCTTCTTCTTCTTCTTCTTCTTCTTCTTCTTCCGGTTGGGTCGGCATGGCATC | DNA | Fill-in with HDVrt (as Ba primer) and *in vitro* transcribe |
| scGAA12 | GATCGATCTCGCCCGCGAAATTAATACGACTCACTATAGGCAGTTCTTCTTCTTCTTCTTCTTCTTCTTCTTCTTCTTCCGGTTGGGTCGGCATGGCATC | DNA | Fill-in with HDVrt (as Ba primer) and *in vitro* transcribe |
| scGAA16 | GATCGATCTCGCCCGCGAAATTAATACGACTCACTATAGGCAGTTCTTCTTCTTCTTCTTCTTCTTCTTCTTCTTCTTCTTCTTCTTCTTCCGGTTGGGTCGGCATGGCATC | DNA | Fill-in with HDVrt (as Ba primer) and *in vitro* transcribe |
| scGAA24 | GATCGATCTCGCCCGCGAAATTAATACGACTCACTATAGGCAGTTCTTCTTCTTCTTCTTCTTCTTCTTCTTCTTCTTCTTCTTCTTCTTCTTCTTCTTCTTCTTCTTCTTCTTCCGGTTGGGTCGGCATGGCATC | DNA | Fill-in with HDVrt (as Ba primer) and *in vitro* transcribe |
| sc8211 | /5Phos/UUC UUC **GCG** UUC UUC **CAG** UUC UUC **UAU** UUC UUC **CAG** | RNA |  |
| A (*8+4 A)*  (termed scA when circularized) | /5Phos/**GGG** UUC UUC **UGG** UUC UUC **UAU** UUC UUC **CAG** UUC UUC | RNA |  |
| scB (*8+4 B)*  (termed scB when circularized) | /5Phos/**CCC** UUC UUC **GUG** UUC UUC **UUU** UUC UUC **CAG** UUC UUC | RNA |  |
| scC *(8+4 C)*  (termed scC when circularized) | /5Phos/**GCC** UUC UUC **GGA** UUC UUC **UAA** UUC UUC **CAG** UUC UUC | RNA |  |
| scD *(8+4 D)*  (termed scD when circularized) | /5Phos/**GCG** UUC UUC **AUC** UUC UUC **GAU** UUC UUC **CAG** UUC UUC | RNA |  |
| scHHz_temp (HHzCtemp_alt7) | /5Phos/GAC CGU UUC GCU CAC GCU CAU CAG GAA CUG GUC CAG UUC | RNA | Template for synthesis of micro HHz |
| **Primers for primer extension assay** |  |  |  |
| F8 | FITC-AA GAA CUG | RNA |  |
| F8+Adap | FITC-CAGUCGUGACCUAAUG-AA GAA CUG | RNA |  |
| P9_1_ (F9) | /56-FAM/GAA GAA CUG | RNA |  |
| P9_1_+Adap (*F9+Adap)* | /56-FAM/CAGUCGUGACCUAAUG-GAA GAA CUG | RNA |  |
| P9 *(F9(GAA))* | /56-FAMN/GAA GAA GAA | RNA |  |
| P9(GAC) *(F9(GAC))* | /56-FAMN/GAC GAC GAC | RNA |  |
| P9(CGG) *(F9(CGG))* | /56-FAMN/CGG CGG CGG | RNA |  |
| P10 *(F10)* | /56-FAM/CUGCCAACCG | RNA |  |
| P10+3 *(10+3GAA)* | /56-FAM/CUGCCAACCG-GAA GAA GAA | RNA |  |
| PHHz (HHz_p12) | GAA C/iFluodT/G GAC CAG | RNA | Primer for HHz circular template |
| **Compeating oligoes** |  |  |  |
| cmp16GAA-p | GATCGATCTCGCCCGCGAAATTAATACGACTCACTATA-GAAGAAGAAGAAGAAGAAGAAGAAGAAGAAGAAGAAGAAGAAGAAGAA-GGGTCGGCATGGCATC | DNA | Fill-in with HDVrt (as Ba primer) and *in vitro* transcribe |
| cmp16GAC-p | GATCGATCTCGCCCGCGAAATTAATACGACTCACTATA-GACGACGACGACGACGACGACGACGACGACGACGACGACGACGACGAC-GGGTCGGCATGGCATC | DNA | Fill-in with HDVrt (as Ba primer) and *in vitro* transcribe |
| cmp16CGG-p | GATCGATCTCGCCCGCGAAATTAATACGACTCACTATA-GGCGGCGGCGGCGGCGGCGGCGGCGGCGGCGGCGGCGGCGGCGGCGGC-GGGTCGGCATGGCATC | DNA | Fill-in with HDVrt (as Ba primer) and in vitro transcribe |
| cmp16GAA | TTCTTCTTCTTCTTCTTCTTCTTCTTCTTCTTCTTCTTCTTCTTCTTCCGGTTGGCAGCUUCCTATAGTGAGTCGTATTAATTTC | DNA | Fill-in with 5T7 (as forward (Fo) primer) and *in vitro* transcribe |
| cmpCTGAA42 | TTCTTCTTCTTCTTCTTCTTCTTCTTCTTCTTCTTCTTCTTCTTCTTCCGGTTGGCAGCUUCCTATAGTGAGTCGTATTAATTTC | DNA | Fill-in with 5T7 (as Fo primer) and *in vitro* transcribe |
| Cmp8211_3ddOx | UGG**G**AGAAAUAGAAGAACUGGAAGAACGCGAAGAA/3ddC/ | RNA |  |
| Cmp8+4A_3ddOx | **UG**GAAGAA**AUA**GAAGAA**CCA**GAAGAA**CCC**GAAGAA /3ddC/ | RNA |  |
| Cmp8+4B_3ddOx | **UG**GGAGAA**AAA**GAAGAA**CAC**GAAGAA**GGG**GAAGAA /3ddC/ | RNA |  |
| Cmp8+4C_3ddOx | **UG**GAAGAA**UUA**GAAGAA**UCC**GAAGAA**GGC**GAAGAA /3ddC/ | RNA |  |
| Cmp8+4D_3ddOx | **UG**GAAGAA**AUC**GAAGAA**GAU**GAAGAA**CGC**GAAGAA /3ddC/ | RNA |  |
| CmpHHz_alt7 | ACCGTTTCGCTCACGCTCATCAGGAACTGGTCCAGTTCTACCGTTTCGCTCACGCTCATCAGGAACTGGTCCAG-CCTATAGTGAGTCGTATTAATTTCGCGGGCGAGATCGATC | DNA | Fill-in with 5T7 (as Fo primer) and *in vitro* transcribe |
|  |  |  |  |
| Splint | CUGCCAACCG | RNA | Splint used for ligation with T4RNA ligase 2 |
| HHzalt7-F | GAA C/iFluroT/G GAC CAG UUC CUG AUG AGC GUG AGC GAA ACG GUC - GAA CUG G | RNA | chemically synthesised self-circularizing micro Hammerhead ribozyme |
| **Oligonucleotides for synthesis of ribozyme polymerase** |  |  |  |
| 5TU (Fo fill-in) | GGATCTTCTCGATCTAACAAAAAAGACAAATCTGCCACAAAGCTTGAGAGCATCTTCGGATGCAGAGGCGGCAGCCTTCGGTGGCGCGATAGCGCCAACGTTCTCAACTATGACACGCAA | DNA |  |
| 5TU (Ba fill-in) | CTTCTCCCTTAGCCTACCGAAGTAGCCCAGGTCGGACCGCGAGGAGGTGGAGATGCCATGCCGACCCCATGATAAACTCCATTCAACGGAGCACGCGTTTTGCGTGTCATAGTTGAGAAC | DNA |  |
| t1 (Fo fill-in) | GACCAATCTGCCCTCAGAGCTCGAGAACATCTTCGGATGCAGAGGAGGCAGGCTTCGGTGGCGCGATAGCGCCAACGTCCTCAACCTCCAATGCATCCCACCACATGATGATGCCTGAAG | DNA |  |
| t1 (Ba fill-in) | CTTCTCCCTTAGCCTACCGAAGTAGCCCAGGTCGGACCGCGAGGAGGTGGAGATGCCATGCCGACCCCAAAAAACCAAGGCTCTTCAGGCATCATCATGTG | DNA |  |
| t5T7pFo | GATCGATCTCGCCCGCGAAATTAATACGACTCACTATAGGATCTTCTCGATCTAAC | DNA |  |
| t1T7pFo | GATCGATCTCGCCCGCGAAATTAATACGACTCACTATAGACCAATCTGCCCTCAG | DNA |  |
|  |  |  |  |
| 5T7 (Fo) | GATCGATCTCGCCCGCGAAATTAATACGACTCACTATA | DNA |  |
| HDVrt (Ba) | CTTCTCCCTTAGCCTACCGAAGTAGCCCAGGTCGGACCGCGAGGAGGTGGAGATGCCATGCCGACCC | DNA |  |
|  |  |  |  |
| 5TU (final RNA product) | GGAUCUUCUCGAUCUAACAAAAAAGACAAAUCUGCCACAAAGCUUGAGAGCAUCUUCGGAUGCAGAGGCGGCAGCCUUCGGUGGCGCGAUAGCGCCAACGUUCUCAACUAUGACACGCAAAACGCGUGCUCCGUUGAAUGGAGUUUAUCAUG | RNA | To make: Fill-in with 5TU Fo and Ba fill-in primers then PCR with t5T7pFo and HDVrt |
| t1 (final RNA product) | GACCAAUCUGCCCUCAGAGCUCGAGAACAUCUUCGGAUGCAGAGGAGGCAGGCUUCGGUGGCGCGAUAGCGCCAACGUCCUCAACCUCCAAUGCAUCCCACCACAUGAUGAUGCCUGAAGAGCCUUGGUUUUUUG | RNA | To make: Fill-in with t1 Fo and Ba fill-in primers then PCR with t1T7pFo and HDVrt |
| **Oligonucleotides for extension product sequencing** |  |  |  |
| PCRp3 (Fo): | CAGTCGTGACCTAATG | DNA |  |
| RTp1 (ba): | CCGTACGGATATTCGAC | DNA |  |
| Adap1: | pGTCGAATATCCGTACG-SpcC3 | DNA | Adap1 was 5'adenylated for later adaptor ligation |
| pGEM_T7_Fo: | GTAATACGACTCACTATAGGGC | DNA |  |
| pGEM_SP6_Ba: | TTTAGGTGACACTATAGAATACTC | DNA |  |
| Ill_Ba: | CAA GCA GAA GAC GGC ATA CGA GAT GTG ACT GGA GTT CAG ACG TGT GCT CTT CCG ATC CCG TAC GGA TAT TCG AC | DNA |  |
| Ill1_Fo: | AAT GAT ACG GCG ACC ACC GAG ATC TAC ACT CTT TCC CTA CAC GAC GCT CTT CCG ATC T - NNN – CTGGAC GCAGUCGUGACCUAAUG | DNA |  |
| Ill2_Fo: | AAT GAT ACG GCG ACC ACC GAG ATC TAC ACT CTT TCC CTA CAC GAC GCT CTT CCG ATC T - NNN – ATACCT GCAGUCGUGACCUAAUG | DNA |  |
| Ill3_Fo: | AAT GAT ACG GCG ACC ACC GAG ATC TAC ACT CTT TCC CTA CAC GAC GCT CTT CCG ATC T - NNN – GTAGCG GCAGUCGUGACCUAAUG | DNA |  |
| Ill4_Fo: | AAT GAT ACG GCG ACC ACC GAG ATC TAC ACT CTT TCC CTA CAC GAC GCT CTT CCG ATC T - NNN – CGCTCC GCAGTCGTGACCTAATG | DNA |  |
| Ill5_Fo: | AAT GAT ACG GCG ACC ACC GAG ATC TAC ACT CTT TCC CTA CAC GAC GCT CTT CCG ATC T - NNN – TATCTT GCAGTCGTGACCTAATG | DNA |  |
| Ill6_Fo: | AAT GAT ACG GCG ACC ACC GAG ATC TAC ACT CTT TCC CTA CAC GAC GCT CTT CCG ATC T - NNN – AGCTAC GCAGTCGTGACCTAATG | DNA |  |
| Ill7_Fo: | AAT GAT ACG GCG ACC ACC GAG ATC TAC ACT CTT TCC CTA CAC GAC GCT CTT CCG ATC T - NNN – CCACCA GCAGTCGTGACCTAATG | DNA |  |
| Ill8_Fo: | AAT GAT ACG GCG ACC ACC GAG ATC TAC ACT CTT TCC CTA CAC GAC GCT CTT CCG ATC T - NNN – AGGATA GCAGTCGTGACCTAATG | DNA |  |
| Ill9_Fo: | AAT GAT ACG GCG ACC ACC GAG ATC TAC ACT CTT TCC CTA CAC GAC GCT CTT CCG ATC T - NNN – ACAAGT GCAGTCGTGACCTAATG | DNA |  |
| Ill10_Fo: | AAT GAT ACG GCG ACC ACC GAG ATC TAC ACT CTT TCC CTA CAC GAC GCT CTT CCG ATC T - NNN – TACTGT GCAGTCGTGACCTAATG | DNA |  |
| Ill11_Fo: | AAT GAT ACG GCG ACC ACC GAG ATC TAC ACT CTT TCC CTA CAC GAC GCT CTT CCG ATC T - NNN – ATTAAC GCAGTCGTGACCTAATG | DNA |  |
| Ill12_Fo: | AAT GAT ACG GCG ACC ACC GAG ATC TAC ACT CTT TCC CTA CAC GAC GCT CTT CCG ATC T - NNN – CACTAT GCAGTCGTGACCTAATG | DNA |  |
| Ill13_Fo: | AAT GAT ACG GCG ACC ACC GAG ATC TAC ACT CTT TCC CTA CAC GAC GCT CTT CCG ATC T - NNN – TGTCGC GCAGTCGTGACCTAATG | DNA |  |
| Ill14_Fo: | AAT GAT ACG GCG ACC ACC GAG ATC TAC ACT CTT TCC CTA CAC GAC GCT CTT CCG ATC T - NNN – ACAGTG GCAGTCGTGACCTAATG | DNA |  |
| Ill15_Fo: | AAT GAT ACG GCG ACC ACC GAG ATC TAC ACT CTT TCC CTA CAC GAC GCT CTT CCG ATC T - NNN – AGCGCC GCAGTCGTGACCTAATG | DNA |  |
